# Inverse Design of Pre‐Strain via a Predictive Electromechanical Model for Enhanced Strain Sensing

**DOI:** 10.1002/advs.77146

**Published:** 2026-08-29

**Authors:** Jia‐Chen Shang, Rui Wang, Bo Lu, Ming‐Liang Song, Yu‐Yi Chen, Kuo Han, Xin‐Jie Feng, Bin Sun, Yao‐Guang Cao, Xiao‐Yu Yan, Shi‐Chun Yang

**Affiliations:** ^1^ School of Transportation Science and Engineering Beihang University Beijing P. R. China; ^2^ Shandong Linglong Tyre Co., Ltd. Zhaoyuan Shandong P. R. China; ^3^ Hangzhou International Innovation Institute Beihang University Hangzhou P. R. China; ^4^ State Key Lab of Intelligent Transportation System Beijing P. R. China; ^5^ Research Institute of Aero‐Engine Beihang University Beijing P. R. China; ^6^ Innovation Center of New Energy Vehicle Digital Supervision Technology and Application for State Market Regulation Beijing P. R. China

**Keywords:** electromechanical modeling, inverse design, microcrack structures, pre‐strain engineering, smart‐tire sensors

## Abstract

Pre‐strain engineering is widely employed to enhance the sensitivity of flexible piezoresistive sensors, yet empirical pre‐straining inevitably triggers severe trade‐offs among sensitivity, linearity, hysteresis, and effective working range, leaving the identification of the optimal pre‐strain an unresolved challenge. Here, an analytical electromechanical model rooted in microcrack evolution mechanics is developed that unifies two governing regimes — conductive‐network fragmentation during initial loading and reversible crack opening–closure during steady‐state cycling — reproducing experimental data with *R* ^2^ > 0.99 across eight pre‐strain levels. Critically, all model parameters reduce to pre‐strain‐independent material constants, leaving strain and pre‐strain as the only inputs; this separation extends the model from per‐specimen fitting to a globally calibrated predictive framework that generates the complete electromechanical response at any untested pre‐strain from only eight calibration datasets — a predictive capability absent from all prior microcrack circuit models. The predictive response surface is then coupled with multi‐objective Pareto optimization to identify the optimal pre‐strain. For smart‐tire monitoring at 10% working strain, the inversely designed sensor (43.0% pre‐strain) reduces median baseline drift from 8.52% to 1.93% (*p* = 1.19 × 10^−^^1^ ^5^), and improves the signal‐to‐noise ratio (SNR) by 68.8% (*p* = 3.11 × 10^−^^1^ ^1^) across 91 bench‐test conditions.

## Graphical abstract

An analytical electromechanical model rooted in microcrack evolution mechanics is globally calibrated from only eight pre‐strain datasets, enabling prediction of the complete sensing response at untested pre‐strain values within the calibrated design space (*R* ^2^ > 0.99). Coupled with uncertainty‐penalized multi‐objective optimization, the framework inversely identifies the optimal pre‐strain for smart‐tire sensors, validated across 91 operating conditions.

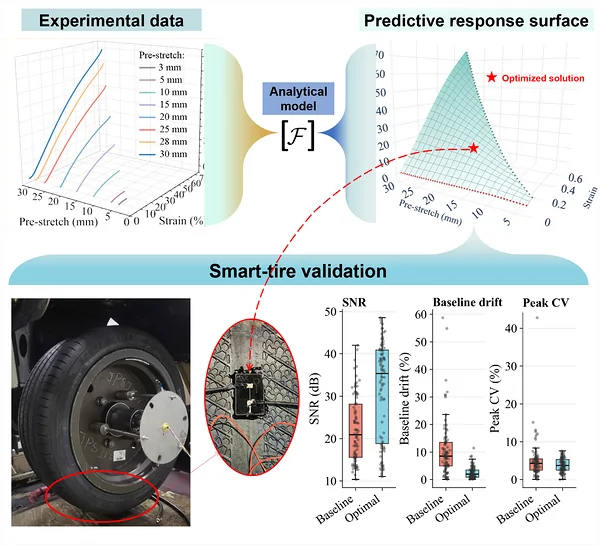

## 1 Introduction

The ongoing convergence of the Internet of Things (IoT) and artificial intelligence [1, 2] has elevated strain‐sensing technology into a critical interface bridging the physical and digital worlds [3, 4, 5, 6]. Among the earliest strain‐sensor architectures [3, 7], piezoresistive sensors remain widely deployed owing to their structural simplicity, low fabrication cost, DC‐compatible (zero‐frequency) response, and straightforward signal conditioning [8]. Rapid advances in flexible electronics have further enabled piezoresistive sensors to combine high sensitivity with broad strain detectability [9, 10], opening applications in artificial skin [11, 12], wearable devices [13, 14], smart tires [15, 16], and intelligent vehicle cabins [17, 18].

Among these scenarios, smart tires impose arguably the most demanding service conditions: a sensor bonded to the tire inner liner must deliver faithful strain signals under high‐frequency cyclic deformation, centrifugal loading, and elevated temperature throughout continuous rolling. Recent prototypes have demonstrated the feasibility of inner‐liner‐bonded flexible strain sensors for estimating acceleration, tire pressure, normal and friction forces, and road surface conditions [15, 16, 19, 20, 21]. However, these efforts focus on downstream estimation algorithms, leaving the upstream design question — how the sensor's structural parameters should be tailored to ensure signal fidelity under dynamic rolling — largely unexamined. In this context, signal fidelity — encompassing baseline stability, cycle‐to‐cycle repeatability, and signal‐to‐noise ratio (SNR) — remains the decisive bottleneck between laboratory demonstration and on‐vehicle deployment.

Advancing sensor performance not only elevates signal quality but also alleviates the complexity and cost of downstream circuitry and algorithms; comprehensive optimization of flexible strain sensors therefore remains a central pursuit in the field. Among the available strategies, structural design offers arguably the most direct and effective route to performance gains [22, 23, 24, 25, 26]. Microcrack architectures, in particular, have attracted broad interest because of their pronounced sensitivity enhancement and facile fabrication [3, 16, 27, 28, 29, 30]. The seminal contribution was made by Kang et al. [31], who drew inspiration from the crack‐shaped slit organs of spider leg joints and introduced microcracks into Pt thin films deposited on elastomeric substrates, realizing an ultrasensitive mechanical sensor with a gauge factor (GF) exceeding 2000 and a detection threshold of 10 nm. Since then, microcrack structures have been widely used to enhance the performance of sensors. Compared with other delicate microarchitectures that demand costly equipment and elaborate processing, microcracks can be readily introduced via pre‐straining [32, 33, 34], fatigue loading [28], or laser scribing [35] — methods that are generally simple, inexpensive, and environmentally benign.

Microcracks, however, are inherently engineered defects: the fragile conductive network that underpins high sensitivity simultaneously entails a narrow effective working range, pronounced nonlinearity, and poor specimen‐to‐specimen reproducibility. Efforts to mitigate these trade‐offs follow two broad strategies. The first reinforces the fragile crack network with a secondary conductive phase: bilayer architectures pairing an ultrasensitive metal film with super‐aligned carbon‐nanotube sheets have achieved 100% stretchability at a GF of 12 274 [34], and carbon‐nanofiber‐embedded conductive layers have expanded the sensing range from 5% to 120% while attaining 97% linearity [36]. Composite reinforcement of this kind is effective, yet it inevitably sacrifices the fabrication simplicity and low cost that make microcrack structures attractive in the first place. The second strategy tailors the crack morphology itself — for instance, by exploiting elastic‐modulus mismatch to induce strain localization and thereby stabilize crack topologies at the critical boundary between network‐ and channel‐type patterns, co‐optimizing sensitivity, stretchability, and linearity [29]. Morphology engineering, however, involves a prohibitively large number of coupled control parameters, rendering explicit parameter‐to‐performance mappings intractable.

Pre‐straining constitutes the most prevalent route to microcrack generation; since it is already integral to the fabrication process, tuning its magnitude to shape the crack architecture offers a performance lever that — unlike the strategies above — introduces no additional process complexity or cost. It is well established that the pre‐strain magnitude governs the density, size, and spacing of the resulting cracks [31, 37, 38, 39], which in turn dictate the electromechanical response. Some studies have compared the effects of several discrete pre‐strain levels on conductive‐layer morphology and sensing performance without pursuing an optimum [37, 38]; others have explicitly sought optimal values, whether through bending‐curvature‐controlled pre‐cracking [31] or uniaxial pre‐stretching, with optima reported at 6% [33] and 10% [39] strain; yet these efforts share a common forward workflow: fabricate several discrete pre‐strain levels, test each, and select the best performer. This trial‐and‐error paradigm suffers from a dual incompleteness. The parameter space is sampled at only a handful of discrete points, and the performance space is collapsed onto a single figure of merit — almost invariably sensitivity — even though sensing performance is intrinsically multidimensional, spanning sensitivity, linearity, working range, hysteresis, and cyclic stability. The pre‐strain values so identified are consequently far from globally optimal.

The persistence of trial‐and‐error design has a deeper root: the quantitative relationship between pre‐strain and sensing performance remains unestablished, because no electromechanical model accepts pre‐strain as a direct input. Existing circuit models for microcracked flexible sensors are formulated exclusively in terms of pre‐existing crack features and, according to crack morphology [3], fall into three categories: through‐channel crack models [31], non‐through‐channel crack models [28], and network crack models [38, 40]. The through‐channel and non‐through‐channel formulations invoke a disconnection–reconnection mechanism built on statistical error functions, leaving several model parameters without clear physical meaning; the network crack model reduces the entire conductive layer to a single minimal sensing unit — a mathematically unjustified simplification — and establishes no quantitative strain dependence for the island and bridge resistances, confining its utility to qualitative interpretation. More fundamentally, any model intended to capture the effect of pre‐strain must describe the complete microcrack evolution from nucleation through propagation; yet all existing formulations presuppose a fully formed crack structure, severing the causal link between pre‐strain and the resulting microstructure. Current microcrack electromechanical models are therefore post‐hoc fitting tools — they interpret measured responses rather than predict them — and consequently offer little guidance for the rational selection of pre‐strain.

To close this gap, this work establishes an analytical electromechanical model that explicitly incorporates pre‐strain as a direct input and in which every parameter is anchored to a defined physical mechanism. This physical grounding reduces all calibratable parameters to pre‐strain‐independent material constants, leaving pre‐strain and working strain as the only input variables. Joint calibration against eight pre‐strain datasets determines a single global parameter set that reproduces all eight electromechanical responses with *R* ^2^ > 0.997. Once these constants are fixed, the model requires no refitting: it generates the complete electromechanical response — a continuous (pre‐strain, working strain, Δ*R*/*R*_0_) surface — at any untested pre‐strain within the calibrated design space. Leave‐one‐out cross‐validation confirms blind‐prediction accuracy above *R* ^2^ = 0.97 for all interior configurations, establishing the model as a genuinely predictive tool rather than a post‐hoc fitting device. From this predictive surface, an inverse‐design database of 10 833 entries is constructed, encompassing working range, sensitivity, linearity, hysteresis, and a composite uncertainty metric; the optimal pre‐strain is identified via uncertainty‐penalized multi‐objective Pareto optimization over the continuous design space — replacing the conventional practice of comparing a handful of discrete specimens against a single figure of merit. A smart‐tire strain sensor targeting a 10% working range serves as the engineering validation: across 91 bench‐test conditions, the inversely designed sensor delivers markedly higher signal quality than the untreated baseline. Collectively, this work converts pre‐strain from an empirically tuned processing parameter into a quantitatively designable variable, establishing a physics‐driven inverse‐design framework for high‐performance flexible piezoresistive sensors.

## 2 Experimental Methods

### 2.1 Materials and Specimen Fabrication

Polyimide (PI) tape served as the carbon precursor; a laser marking machine (AtomStack, P1) generated a porous conductive layer in situ on its surface. Polydimethylsiloxane (PDMS, Sylgard 184, Dow Corning) was infiltrated into the porous layer and subsequently cured at elevated temperature, yielding a composite conductive structure with stable mechanical, and electrical properties. Electrical connections between the conductive layer and external leads were established with conductive silicone adhesive (TH‐7003, Shenzhen Watihe Technology Co., Ltd., China).

### 2.2 Tensile Testing and Resistance Acquisition

Type 2 dumbbell specimens (thickness 1.2 mm) were prepared in accordance with ISO 37:2017(E) for all pre‐strain studies. Pre‐stretching and every subsequent mechanical test were conducted under controlled laboratory conditions on a universal testing machine (Instron 5966) at a crosshead speed of 20 mm min^−^^1^ unless otherwise stated. Strain was monitored in real time with a video extensometer, while resistance was recorded synchronously via a dynamic data acquisition system (DH5902N).

Because pre‐strains approaching the fracture limit can introduce substantial micro‐damage that degrades the electrical response and shortens service life, the maximum pre‐stretching displacement was capped at 30 mm (nominal strain 71.6%). Eight pre‐stretch levels, spanning applied displacements from 3 to 30 mm, were selected to cover the feasible design space systematically. Because the gauge‐section strain scales linearly with the applied displacement Section 3.1, Figure S1, each pre‐stretch level maps one‐to‐one onto a nominal pre‐strain; Table 1 lists this correspondence for the eight groups investigated. For conciseness, specimens are hereafter designated by their pre‐stretch displacement (e.g., “3 mm pre‐stretch”). 20 loading–unloading cycles were executed at each level to suppress stochastic scatter and confirm repeatability.

**TABLE 1:** Correspondence between applied pre‐stretch displacement and nominal pre‐strain for the eight specimen groups investigated in this study.

Pre‐stretch displacement (mm)	Pre‐strain (%)
3	7.16
5	11.93
10	23.87
15	35.81
20	47.74
25	59.65
28	66.83
30	71.60

### 2.3 Microstructural Characterization

In situ microstructural evolution during stretching was captured with a scanning electron microscope (Apreo S LoVac, Thermo Fisher Scientific) fitted with a miniature tensile stage. Type 3 dumbbell specimens, fabricated following ISO 37 with a process identical to that of the Type 2 specimens, were employed to comply with the spatial constraints of the stage. The in situ loading rate was 1 mm min^−^^1^; the maximum attainable displacement was 13 mm (nominal strain 40.9%), governed by the stage travel. To convert the applied displacement into the local strain at the observation zone, a finite element model of the Type 3 dumbbell was established in Abaqus under a 13 mm displacement load, providing a displacement‐to‐local‐strain mapping for accurate calibration at each loading increment.

### 2.4 Tire Bench Testing

Sensor coupons (30 mm × 20 mm × 1 mm) were fabricated with the same process as the tensile specimens, differing only in mold geometry. Each coupon was then pre‐stretched on the universal testing machine to the optimal pre‐strain magnitude identified by the inverse‐design framework. Both pre‐stretched and baseline (non‐pre‐stretched) specimens were bonded along the centerline of the inner tread surface of 235/45R18 tires; the bonding area was lightly abraded beforehand to promote adhesion, and 3M RT5000B adhesive was applied. The instrumented tires were mounted on a tire force‐and‐moment test rig (MTS FLAT‐TRAC III CT) for bench evaluation. Sensor output was acquired in differential‐voltage mode through a series voltage‐divider circuit using a DH 5902N dynamic signal acquisition system.

## 3 Results and Discussion

### 3.1 Mechanical Characteristics

Before investigating the effects of pre‐strain, uniaxial tension‐to‐failure tests were conducted to establish the mechanical limits and sensing range of the specimens. As shown in Figure S1, the nominal strain in the gauge section of the Type 2 dumbbell specimen scales linearly with the crosshead displacement, and fracture occurs at a displacement of 34.2 mm. The corresponding stress–strain curve Figure 1a reveals pronounced hyperelastic behavior up to a fracture strain of 81.7% and a fracture stress of 2146.7 kPa. A first‐order Ogden model (μ_1_ = 528.8 kPa, α_1_ = 4.77) captures this response; the fitted parameters were imported into Abaqus and used to simulate the tensile deformation up to 34 mm displacement. The simulated data, overlaid as discrete markers in Figure 1a, agree closely with the experimental curve (*R* ^2^ = 0.998), confirming that the first‐order Ogden formulation adequately describes the nonlinear elasticity of the PDMS matrix. Figure 1b tracks the normalized resistance change, defined as Δ*R*/*R*_0_ = (*R* − *R*_0_)/*R*_0_ with *R*_0_ the quiescent resistance, as a function of tensile strain. The piezoresistive response persists over the full stretch range up to structural fracture — the sensing‐limit strain exceeds the fracture strain — indicating that the sensor's electrical detectability spans the entire usable deformation window of the substrate.

**FIGURE 1:**
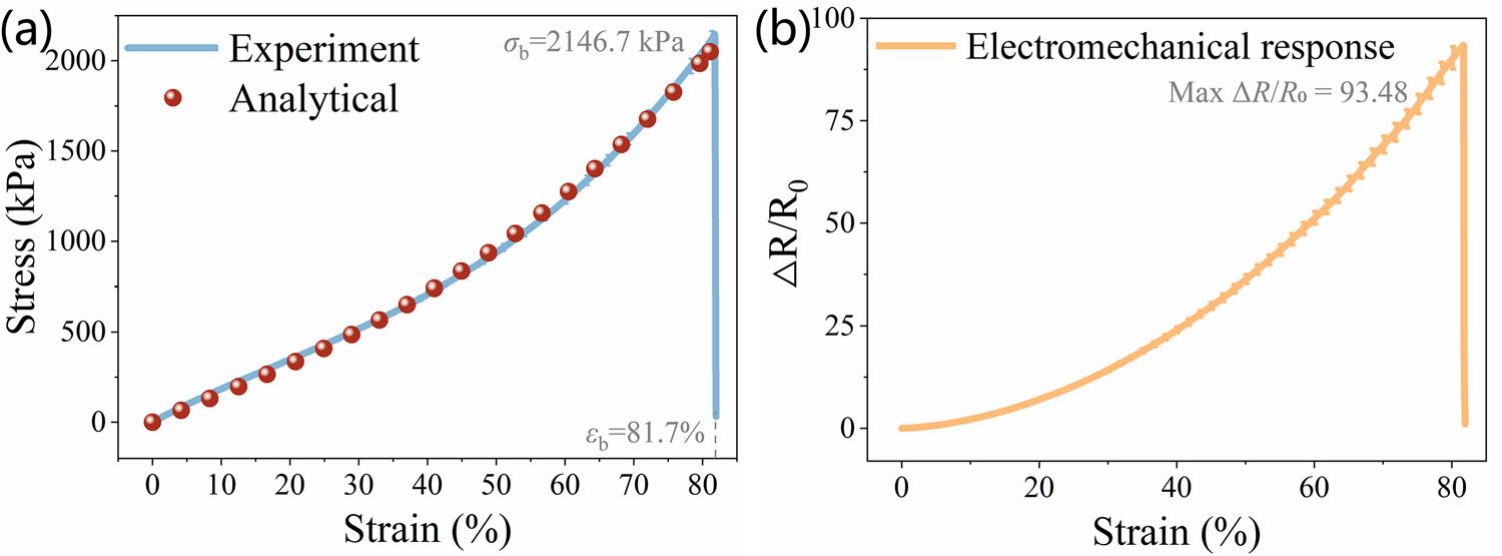
Fundamental characterization of the pristine sensor. (a) Uniaxial stress–strain curve with first‐order Ogden model fit. (b) Relative resistance change (Δ*R*/*R*_0_) recorded monotonically until mechanical fracture.

### 3.2 In Situ Evolution of Microcrack Structures

Figure 2a presents in situ scanning electron microscopy (SEM) micrographs captured over a complete loading–unloading cycle. During loading Figure 2a_1_–a_5_, the conductive layer surface at 0% strain Figure 2a_1_ displays only the intrinsic groove texture inherited from laser processing, with no discernible cracks. At 13% strain Figure 2a_2_, a dense population of microcracks has nucleated perpendicular to the tensile direction, adopting a quasi‐periodic parallel arrangement. Further stretching to 22.6% and 31.9% Figure 2a_3_,a_4_ progressively increases both the crack density and the individual crack dimensions, steadily narrowing the island‐shaped conductive regions that separate adjacent cracks. At the peak strain of 40.9% Figure 2a_5_, the cracks have fully developed and locally coalesced into wide fissures, leaving the conductive network in a highly fragmented state. The subsequent unloading sequence Figure 2a_5_–a_9_ reveals partial structural reversibility: crack widths contract markedly and some opposing edges re‐establish contact, yet the cracks themselves persist. At full unload Figure 2a_9_, residual crack traces remain clearly visible, confirming that the damage introduced during first loading is irreversible. This loading–unloading asymmetry underpins the distinction between the initial pre‐cracking phase and the subsequent steady‐state working phase discussed in later sections.

**FIGURE 2:**
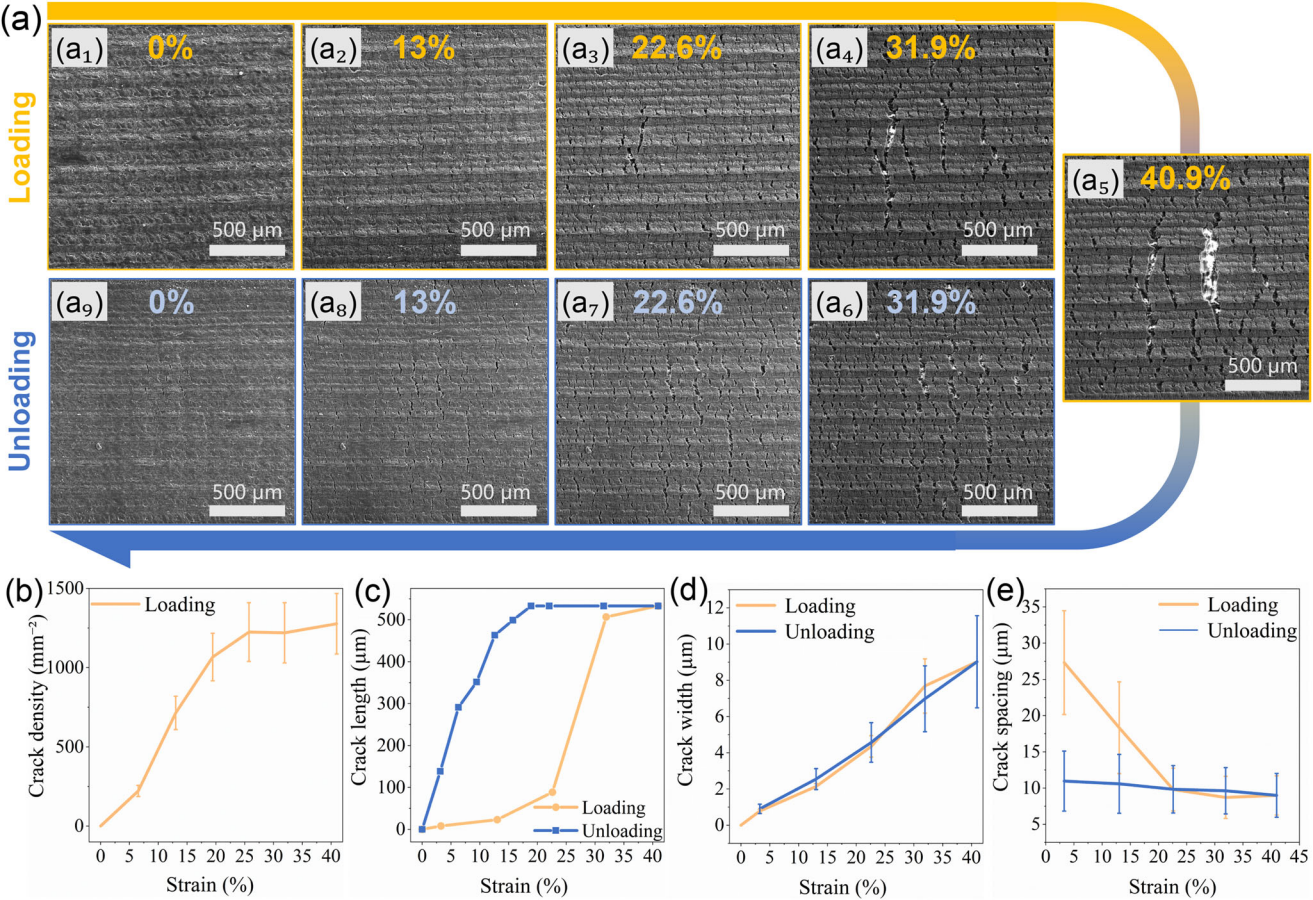
In situ SEM characterization of microcrack evolution during the first loading–unloading cycle. (a) Representative SEM micrographs (scale bars: 500 µm) of the conductive layer at selected strain levels; upper row (a_1_–a_5_): loading; lower row (a_6_–a_9_): unloading. (b) Crack areal density vs. strain. (c) Individual crack length during loading and unloading. (d) Maximum crack width vs. strain. (e) Crack spacing vs. strain.

Quantitative crack statistics were extracted from the SEM images using ImageJ (FIJI) and are summarized in Figure 2b–e.

The areal crack density during loading Figure 2b follows a characteristic two‐stage pattern: a rapid, approximately linear increase at low strains (0%–25%), and followed by saturation beyond ∼25% strain. This plateau indicates that the available stress‐concentration sites within the thin conductive film have been exhausted, consistent with previous reports [32, 41, 42]. The density evolution is well captured by an exponential saturation law:

(1) $$n=N_{sat}\left[1-\exp\left(-k_{n}\cdot \varepsilon \right)\right]$$

where *N*_sat_ denotes the saturation crack density and *k*_n_ the proliferation rate constant.

Characterizing crack length through conventional ensemble averaging is problematic for two reasons: cracks span multiple length scales and do not nucleate synchronously, so averaging masks the underlying size distribution; moreover, during unloading, edge re‐contact morphologically segments a single continuous crack into several shorter fragments, producing a non‐physical drop in the mean length. We therefore track a single representative crack throughout the cycle Figure 2c. During loading (orange curve), three distinct regimes emerge — a slow incubation phase at low strains, a rapid propagation phase once a critical strain threshold is exceeded, and a saturation phase governed by the fracture toughness and macroscopic geometry of the film. Upon unloading (blue curve), the tracked crack initially retains its full length; only at lower strains does the apparent length decrease sharply as narrowing crack faces re‐contact and visually subdivide the crack. Crucially, this apparent shortening is a morphological artifact of re‐bridging rather than genuine crack healing.

The maximum width of an individual crack evolves nearly linearly with strain during both loading and unloading Figure 2d, and the two paths overlap closely. This high degree of reversibility indicates that the crack‐opening displacement is governed predominantly by the elastic deformation of the PDMS substrate, with a minor hysteresis attributable to the viscoelastic character of the matrix.

The spacing between adjacent cracks — equivalently, the characteristic island dimension [34, 38, 40] — is a key geometric parameter controlling the macroscopic conductivity of the cracked conductive layer. As quantified in Figure 2e, the island size undergoes a steep decline in the low‐strain regime, mirroring the concurrent surge in crack density as newly nucleated cracks repeatedly bisect existing islands. Once the crack population saturates, the spacing stabilizes. Importantly, the island dimension remains essentially invariant throughout unloading, demonstrating that microscale strain recovery is accommodated entirely by reversible crack‐width closure while the island topology is preserved intact — an observation consistent with earlier findings [38]. This geometric stability furnishes direct experimental justification for treating the island array as a fixed structural unit in the electromechanical model developed in Section 3.4.

### 3.3 Electromechanical Behavior of Pre‐Strained Specimens

Although pre‐straining is widely recognized for its pronounced effect on the electromechanical behavior of flexible piezoresistive sensors [3, 39], the quantitative relationship between pre‐strain magnitude, and the resulting electromechanical response remains elusive. To map this relationship systematically, we tracked the full electromechanical evolution of eight specimen groups over 20 consecutive tensile cycles, thereby furnishing the physical basis for an electromechanical model that explicitly incorporates pre‐strain.

Figure 3a shows the time‐domain resistance trace of a 20 mm pre‐stretch specimen across the first few cycles. The first cycle is distinctly transient: the peak resistance change reaches Δ*R*/*R*_0_ = 33.20 and the loading–unloading paths are markedly asymmetric. From the second cycle onward, the response stabilizes rapidly, with the peak converging to a higher value (Δ*R*/*R*_0_ = 36.56) and negligible inter‐cycle variation. The counter‐intuitive observation that the first‐cycle peak lies below the steady‐state peak has a clear physical origin: initial loading begins from an intact conductive layer and a fraction of the strain range is consumed by crack nucleation, whereas steady‐state cycles depart from a pre‐cracked state in which the entire strain range drives reversible crack opening, thereby generating a larger resistance change at the same nominal strain. A residual resistance of Δ*R*/*R*_0_ = 1.37 is recorded upon the first full unload. Although cracks appear morphologically closed at 0% strain Figure 2a_9_, contact resistance at partially re‐mated crack faces permanently elevates the layer resistivity above its pristine value. This residual stabilizes at Δ*R*/*R*_0_ ≈ 1.36 from the second cycle onward, signaling that the crack‐network topology has reached dynamic equilibrium. These observations suggest a fundamental shift in the governing electromechanical mechanism between the first cycle and subsequent cycles. The resistance–strain hysteresis loops in Figure 3b provides direct evidence for this mechanistic shift. The initial loading curve (red) exhibits characteristic concave nonlinearity — *d*(Δ*R*/*R*_0_)/*dε* increases monotonically with strain — reflecting the progressive fragmentation of the conductive network through irreversible crack nucleation and propagation. The initial unloading path (gray) is convex. From the second cycle onward (blue), both branches adopt a convex form with markedly reduced loop area, confirming that the dominant mechanism has shifted to the reversible opening and closure of pre‐existing cracks. We accordingly define cycle 1 as the “initial loading–unloading interval” and subsequent cycles as “steady‐state cycles”. Because the steady‐state characteristics are rooted in the crack topology established during the first cycle, dissecting the first‐cycle electromechanics is the essential prerequisite for quantifying the role of pre‐strain.

**FIGURE 3:**
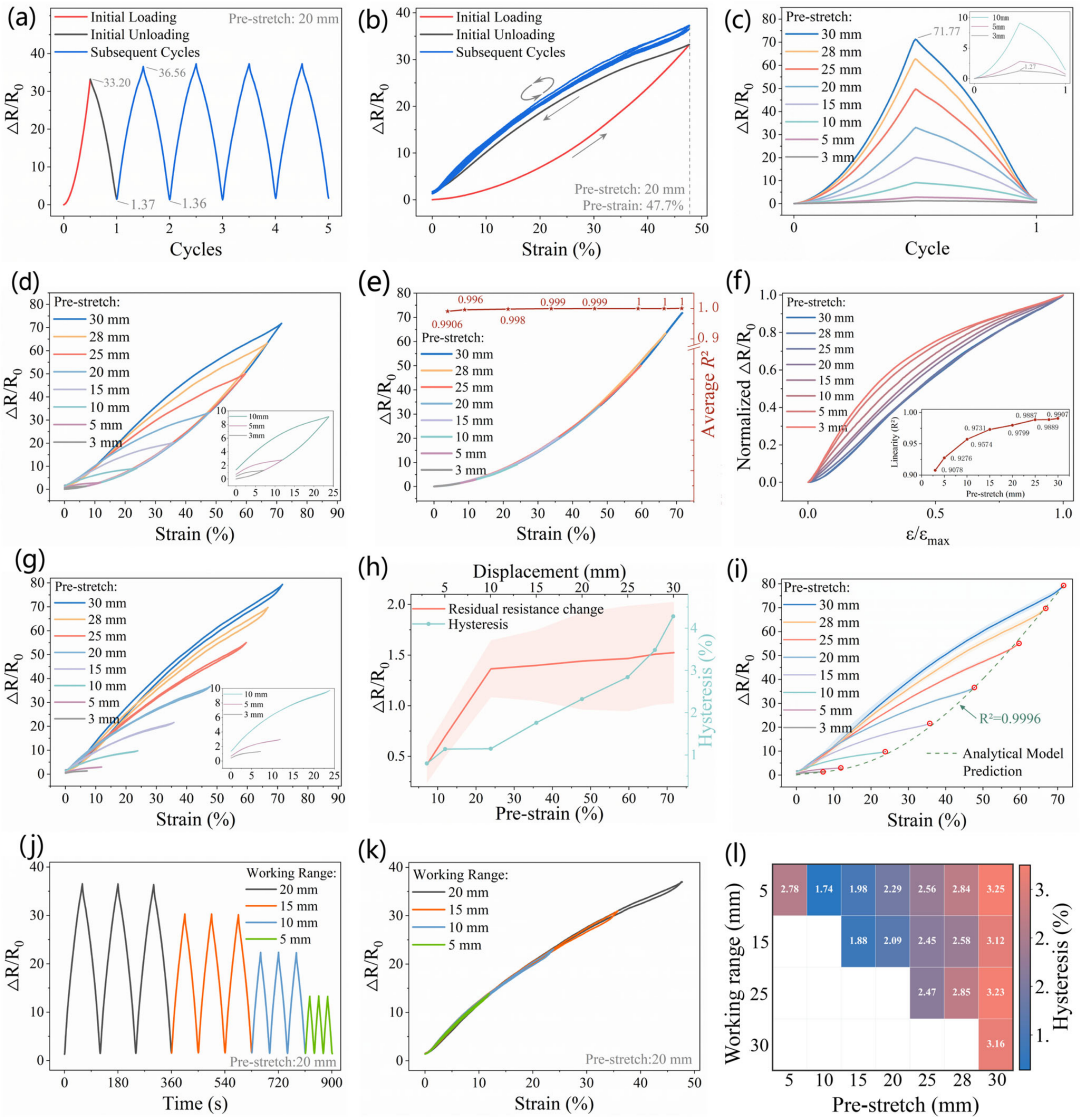
Systematic electromechanical characterization of pre‐strained specimens. (a) Time‐domain resistance evolution over consecutive cycles. (b) Resistance–strain hysteresis loops. (c) First‐cycle responses across all pre‐stretch levels. (d) Superposition of full first‐cycle hysteresis loops. (e) First‐cycle loading trajectories aligned to the 30 mm reference curve, with *R* ^2^ values. (f) Min–max normalized unloading trajectories. (g) Steady‐state hysteresis loops. (h) Residual resistance and hysteresis vs. pre‐stretch displacement. (i) Mean steady‐state responses with error bands, peak responses, and analytical model prediction. (j) Time‐domain response under step‐down sub‐range cycling. (k) Sub‐range resistance–strain hysteresis loops overlaid on the full‐range envelope. (l) Hysteresis heat map across pre‐stretch levels and working sub‐ranges.

Figure 3c compiles the first‐cycle responses of all eight pre‐stretch levels. Despite near‐identical curve morphologies, the peak Δ*R*/*R*_0_ escalates exponentially with pre‐strain — from 1.27 at 3 mm pre‐stretch to 71.77 at 30 mm pre‐stretch. A mere 10‐fold increase in applied displacement produces a nearly 60‐fold increase in resistance change, underscoring the strongly nonlinear leverage of pre‐strain on sensitivity.

The first‐cycle hysteresis loops Figure 3d reveal a further important regularity: the loading branches of all specimens collapse onto a single master curve. Taking the 30 mm loading curve as the baseline and computing the coefficient of determination against every other group over the corresponding strain window yields *R* ^2^ > 0.99 for all cases Figure 3e. This universal overlap carries a decisive modeling implication — the first‐cycle loading curve of the most heavily pre‐strained specimen serves as a single master function that describes the loading‐phase electromechanics at any pre‐strain level.

To compare unloading‐branch morphologies, whose absolute magnitudes differ by orders of magnitude, each specimen's strain and Δ*R*/*R*_0_ data were subjected to min–max normalization Figure 3f. All normalized unloading curves share a convex envelope, with linearity improving systematically as pre‐strain increases (*R* ^2^ rising from 0.9078 at 3 mm pre‐stretch to 0.9879 at 30 mm pre‐stretch). Furthermore, the Pearson correlation heat map Figure S2, minimum *r* > 0.97) statistically validates the strong intrinsic morphological similarity across all unloading paths. This universal geometric similarity confirms that a single, consistent micromechanical mechanism governs the unloading electromechanical behavior across the entire pre‐strain spectrum, thereby establishing a rigorous physical premise for the analytical electromechanical model developed in the following section.

Figure 3g presents the averaged steady‐state hysteresis loops. Both the loading and unloading branches are convex, and the enclosed loop area — a direct measure of hysteresis — grows visibly with increasing pre‐strain. Figure 3h quantifies this trend: hysteresis (right axis) rises monotonically from 0.42% (3 mm specimen) to 4.28% (30 mm specimen), a 10‐fold span that highlights the strong regulatory role of pre‐strain. The residual resistance (left axis) follows a two‐stage pattern: a steep, approximately linear increase for pre‐stretch displacements below 10 mm, followed by a gradual plateau. Because residual resistance reflects the cumulative electrical footprint of structural damage, this two‐stage behavior mirrors the crack‐density evolution reported in Figure 2b — rapid proliferation at low strains driving a sharp resistance rise, followed by crack saturation and a leveling‐off — thereby forging a direct link between macroscopic electrical metrics and microscale structural features.

The mean steady‐state response curve and associated error band for each pre‐strained specimen are obtained by averaging the loading and unloading branches of the steady‐state hysteresis loop Figure 3g at each strain level; the result is plotted in Figure 3i. As established in Figure 3a, the peak resistance change during the first cycle is consistently lower than that of the subsequent steady‐state cycles, the red markers in Figure 3i accordingly denote the steady‐state peak response (maximum Δ*R*/*R*_0_) for each group. These peak values were subsequently input into the analytical electromechanical model developed in this study for parameter inversion fitting, yielding an exceptionally high fitting accuracy (*R* ^2^ = 0.9991).

In practice, a sensor's working strain is typically below its pre‐strain value, making sub‐range dynamic fidelity a critical performance requirement. We therefore cycled a representative 20 mm pre‐stretch specimen at step‐decreasing displacement amplitudes of 20, 15, 10, and 5 mm. The time‐domain resistance profiles Figure 3j confirm stable periodic behavior across all sub‐ranges. When the same data are replotted as resistance–strain hysteresis loops Figure 3k, every sub‐range trajectory collapses onto the master envelope defined by the full pre‐strain, with no detectable offset.

This result establishes a robust structural memory effect: the initial pre‐stretching operation defines the ultimate physical boundary of the crack network, and any subsequent deformation smaller than the pre‐strain modulates resistance exclusively through reversible opening and closure of existing cracks, without inducing new microstructural damage. The sensor's sub‐range dynamics are therefore strictly and predictably governed by its pre‐strain history — a deterministic relationship that provides the mechanistic basis for incorporating pre‐strain as the primary design variable in the electromechanical model developed below. Leveraging this memory, the steady‐state response of any pre‐strained specimen over an arbitrary sub‐range can be extracted directly from its full‐range response profile.

Although the physical origins of hysteresis are complex and preclude direct sub‐range prediction from full‐range data, systematic sub‐range cycling experiments Figure 3l reveal a remarkably simple pattern: under a given pre‐strain constraint, the hysteresis increases moderately with the selected working range, yet the dominant factor governing the hysteresis level is the pre‐strain magnitude rather than the working range itself. For a fixed working range, the hysteresis rises substantially with increasing pre‐strain, confirming that pre‐strain serves as the primary control variable for hysteresis.

### 3.4 Microcrack‐based Electromechanical Model

Under tensile loading, microcracks nucleating within the conductive layer sever current pathways and raise the macroscopic resistance; upon unloading, progressive crack closure restores conduction, and the resistance falls. This qualitative picture, however, cannot account for the markedly different response morphologies observed in the initial loading interval vs. the steady‐state cycles Section 3.3. To resolve this discrepancy quantitatively and derive an analytical resistance–strain relationship, we develop a physics‐based electromechanical model governed by microcrack evolution. The complete step‐by‐step derivation is provided in Note S3; the main text presents the core physical assumptions, the key intermediate relation on which the global calibration of Section 3.6 rests, and the final governing equations.

Figure 4a traces the abstraction from real morphology to circuit representation. The SEM image Figure 4a–i reveals a network‐type crack pattern [3, 28, 29], which is idealized in Figure 4a‐ii as a periodic array of parallel cracks spanning a conductive layer of length *L* and width *W*. The crack spacing along the tensile direction is *d* = *L*/(*n*+1) and the crack length is *l*_c_. The corresponding circuit model Figure 4a‐iii [34, 38, 40] is built from a minimum repeating unit (red dashed box) whose resistance *R*_u_ comprises three elements: the bridge resistance *R*_1_​ (blue) running parallel to the cracks, the island resistance *R*_2_ (orange) running perpendicular, and the crack‐interface contact resistance *R*_c_​ (green). The unit cell measures *l* in the crack‐length direction and *d* + *w*_c_ in the tensile direction, with *w*_c_ the crack width.

**FIGURE 4:**
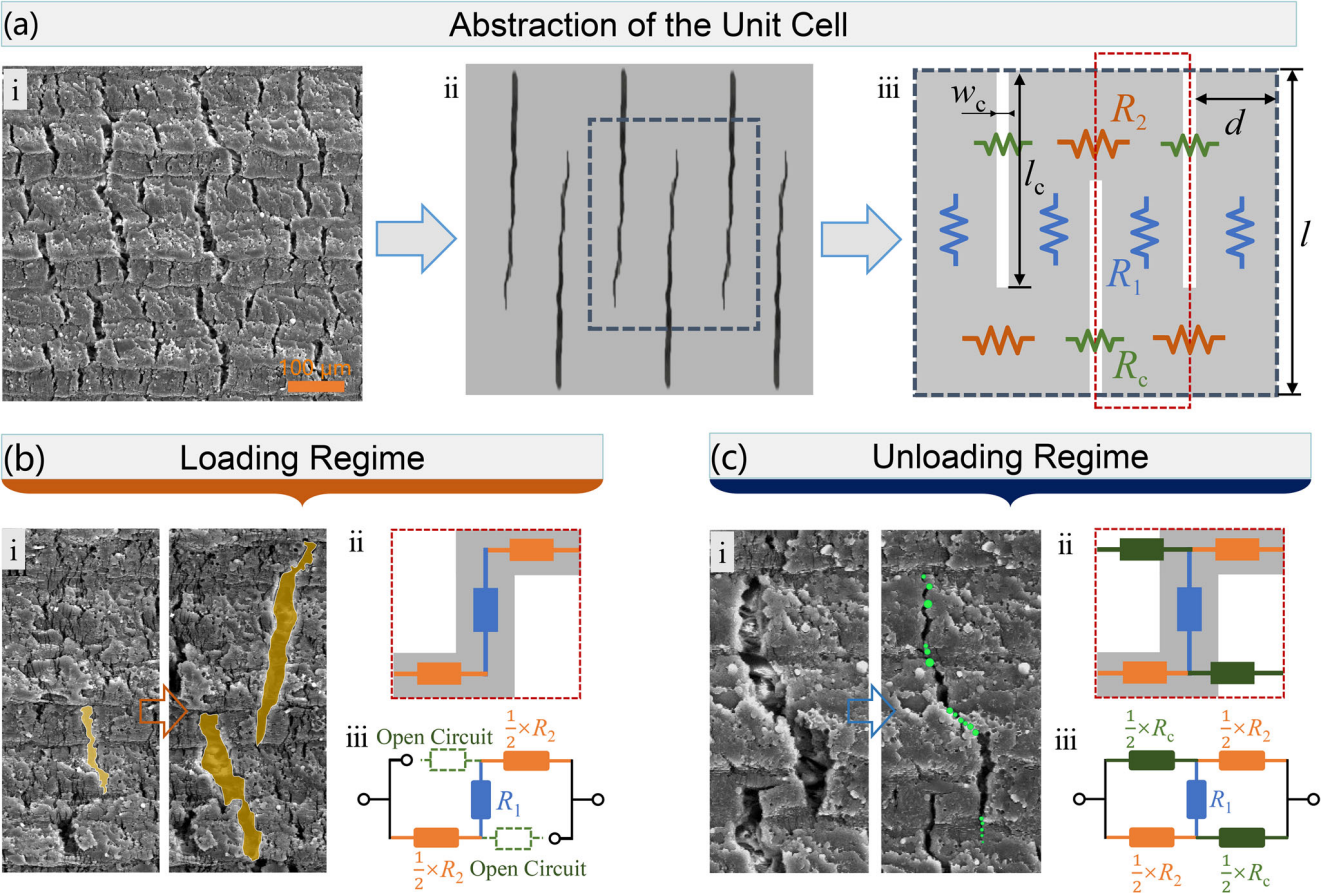
Construction of the microcrack‐based electromechanical model. (a) Abstraction from real morphology to circuit representation: (i) SEM image showing network‐type cracks in the conductive layer; (ii) idealized crack network; (iii) equivalent circuit model of the representative unit cell. (b) Loading regime: (i) in situ SEM images showing rapid crack opening (highlighted in orange); (ii) schematic of the unit cell during loading; (iii) corresponding circuit model with the crack treated as an open circuit. (c) Unloading regime: (i) in situ SEM images showing crack‐width contraction and asperity‐mediated recontact (green markers); (ii) schematic of the unit cell during unloading; (iii) corresponding circuit model with a finite crack contact resistance.

The loading regime rests on a single defining assumption, supplied directly by experiment: in situ SEM images captured during the first stretch Figure 4b–i show that the material on either side of a newly formed crack separates abruptly, leaving no bridging contact. The crack interface is therefore treated as an open circuit throughout initial loading Figures 4b‐ii, iii, and the unit‐cell resistance reduces to the series combination of the bridge and island contributions:

(2) $$R_{u}^{\left(1\right)}=R_{1}+R_{2}$$

Each geometric and material quantity in the unit cell evolves according to an experimentally anchored law: the crack length *l*_c_ follows Gompertz kinetics calibrated against the in situ tracking data of Figure 2c and Equation S5; the crack count *n* obeys the exponential saturation law of Equation (1) and Figure 2b; the crack width *w*_c_ scales linearly with strain as *w_c_* = *k_w_* · ε Figure 2d; and the directional resistivities of the conductive layer are coupled to the applied strain through the constitutive relations of Equations S2 and S4. Assembling the *n*‐unit network, normalizing by the crack‐free initial resistance *R*_0_ Equations S6 and S7, and substituting these relations yields the governing equation of the loading phase:

(3) $$\begin{array}{c} {\left(\frac{\Delta R}{R_{0}}\right)}_{load}=\frac{l\Gamma \left(\lambda_{\varepsilon }+1\right)l_{c}^{sat}T\left(\varepsilon \right){\left[N+1\right]}^{2}}{L^{2}}+\frac{l\phi \left(\varepsilon \right)\left[L+\left(n+1\right)k_{w}\varepsilon \right]}{L\left[l-l_{c}^{sat}T\left(\varepsilon \right)\right]}-1 \end{array}$$

where $\Gamma =\frac{\rho_{1}^{\left(0\right)}}{\rho_{2}^{\left(0\right)}}$ is the ratio of the initial conductivities in the two directions, λ is a coefficient characterizing the degree of influence of tensile strain on the width‐direction resistivity, $l_{c}^{\mathrm{sat}}$ is the saturation crack length, *T*(ε) = *exp*[− *k*_*c*1_] *exp*(− *k*_*c*2_ ε) − *exp*(− *k*_*c*1_), and $\phi \left(\varepsilon \right)=\alpha \varepsilon^{\beta }+1$.

The unloading regime is governed by two experimentally anchored assumptions. First, the crack topology formed during loading is frozen: the maximum crack length $l_{c}^{max}$ and the crack spacing *d* ^fix^ no longer change — corroborated by the constant crack spacing throughout unloading in Figure 2e — so the bridge geometry is fixed at its maximum‐strain configuration Equations S8 and S9, while the island resistance retains its strain dependence through the linearly closing crack width Equation S10. Second, conduction across the crack is progressively restored: as strain decreases, protruding microscopic asperities on the opposing crack faces re‐establish contact (green markers, Figure 4c‐i) — the disconnection–reconnection mechanism identified by Kang et al. [31]. Unlike loading, the crack region now contains re‐bridged portions that conduct current, introducing a finite crack contact resistance *R*_c_ into the unit cell Figure 4c‐ii, iii [38, 40, 43]:

(4) $$R_{u}^{\left(2\right)}=\frac{R_{1}R_{2}+R_{2}R_{c}+R_{1}R_{C}}{4R_{1}+R_{2}+R_{C}}$$

The strain dependence of *R*_c_ follows from statistical contact mechanics: contact is established wherever the local crack width falls below the height of an opposing asperity. According to the Greenwood–Williamson model, asperity heights are exponentially distributed, so the contact area — and hence the normalized crack conductance — varies exponentially with crack opening Equations S11–S13:

(5) $$S_{c}=\frac{1}{1-e^{-k_{S}\cdot \varepsilon_{\max}}}\left(e^{-k_{S}\cdot \varepsilon }-e^{-k_{S}\cdot \varepsilon_{\max}}\right)$$

where *k_s_* is a decay coefficient reflecting crack‐surface roughness; a larger *k_s_* indicates contact concentrated near *ε* = 0. As established in Section 3.6, *k_s_* is the sole model parameter that varies with pre‐strain — reflecting pre‐strain‐induced changes in interfacial roughness — and its explicit parameterization underpins the global calibration. With $R_{c}^{\left(0\right)}$ denoting the contact resistance at full crack closure, the crack resistance at strain *ε* follows as $R_{c}=R_{c}^{\left(0\right)}/S_{c}$.

Because the crack count remains fixed at its maximum‐strain value *n*_max_ = *N_sat_* [1 − *exp*(− *k_n_* · ε_max_)] during unloading, assembling the network Equation S14 and normalizing by *R*_0_ yields the governing equation of the unloading phase:

(6) $$\begin{array}{c} {\left(\frac{\Delta R}{R_{0}}\right)}_{\mathrm{unlo}ad}=\frac{lh}{\rho_{2}^{\left(0\right)}L}\left(n_{max}+1\right)\frac{S_{C}R_{1}^{\left(2\right)}R_{2}^{\left(2\right)}+\left(R_{1}^{\left(2\right)}+R_{2}^{\left(2\right)}\right)R_{c}^{\left(2\right)}}{S_{C}\left(4R_{1}^{\left(2\right)}+R_{2}^{\left(2\right)}\right)+R_{c}^{\left(0\right)}} \end{array}$$

Equations (3) and (6) together constitute the complete analytical description of the electromechanical response: Equation (3) captures the irreversible fragmentation of the conductive network during initial loading, and Equation (6) the reversible opening–closure of the established crack network — the regime governing all steady‐state operation.

### 3.5 Model Validation

Equations (3) and (6) were independently fitted to the first‐cycle electromechanical response of all eight specimen groups; the theoretical curves are compared with the experimental data in Figure S3 (parameters listed in Table S1). Even the lowest‐accuracy group (3 mm) achieves *R* ^2^ = 0.9997, confirming that the analytical equations faithfully capture both the crack nucleation–propagation kinetics during initial loading and the reversible crack‐closure behavior during unloading.

The steady‐state cycles share the same reversible opening–closure mechanism as the first‐cycle unloading, as evidenced by Pearson correlation coefficients exceeding 0.999 between the mean steady‐state response and the first‐cycle unloading curve Figure S4. Therefore, the unloading‐phase model Equation 6 was further applied to fit the mean steady‐state response curves of each specimen Figure S5, yielding *R* ^2^ ≥ 0.9997 for all groups Table S2.

This level of validation, however, constitutes phenomenological fitting: a separate parameter set is calibrated for each specimen, and the model can only *interpret* configurations that have already been tested — it provides no predictive power for untested pre‐strain values.

### 3.6 Global Calibration and Establishment of Predictive Capability

To extend the model from post‐hoc fitting to generalized prediction across untested pre‐strain values, we introduce a global parameter calibration strategy. The underlying physical premise is that the microcrack propagation kinematic parameters ($l_{c}^{sat}$, *k*_*c*1_, *k*_*c*2_, *N_sat_*, *k_n_*) and the conductivity–strain coupling parameters (λ, α, β) represent intrinsic material properties of the conductive composite. These parameters must therefore act as global constants, independent of pre‐strain. Only the crack‐surface attenuation coefficient *k_s_*— which reflects pre‐strain‐induced changes in micro‐interfacial roughness — and the residual resistance $R_{c}^{\left(0\right)}$ are treated as functions of the pre‐strain amplitude.

Based on the strong morphological similarity among the normalized unloading profiles Figure 3f, the raw strain and resistance data were projected into a dimensionless coordinate space (strain: [0, 1]; resistance change: [1, 10]), as depicted in Figure 5a. This normalization removes the magnitude differences caused by varying pre‐strain levels and absorbs the baseline offset of the residual resistance, allowing all nine physical parameters to be constrained as shared global constants. Guided by the systematic trend in linearity with increasing pre‐stretch Figure 3f, inset, the sole pre‐strain‐dependent variable *k_s_* is parameterized as an exponential decay function, *k_s_* = *Aexp*(− *B* · ε_*pre*_) + *C*, introducing three auxiliary coefficients. The model thus achieves mathematical closure with exactly 12 calibratable global parameters and only two physical inputs: pre‐strain and operational strain.

**FIGURE 5:**
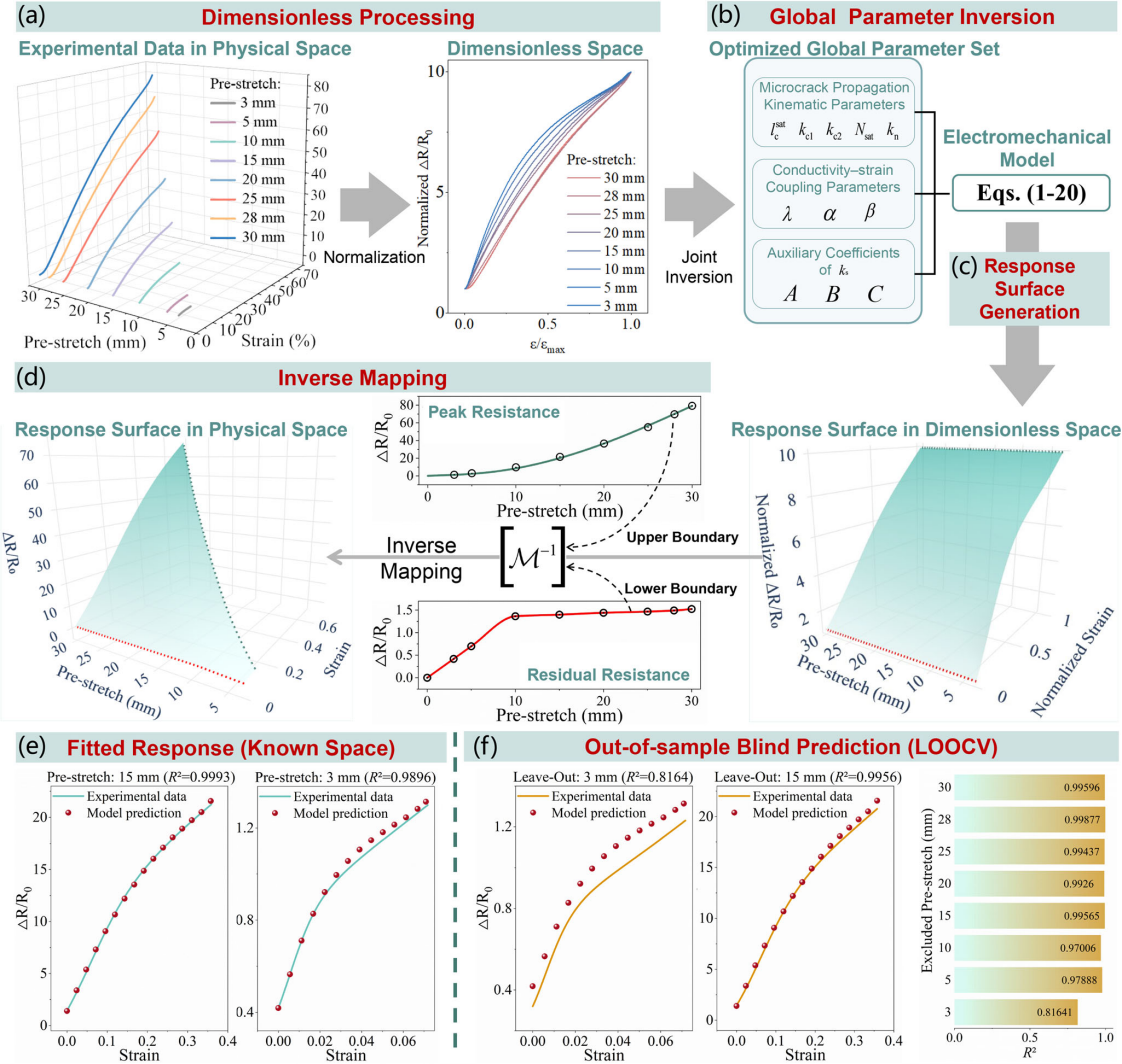
Global parameter calibration and predictive validation of the analytical model. (a) Dimensionless mapping of experimental hysteresis profiles from physical space to normalized strain–response space. (b, c) Joint inversion of dimensionless datasets to obtain the global parameter set and generate the analytical response surface. (d) Inverse mapping to physical space using peak resistance and residual resistance as boundary constraints. (e) Model fitting within the known parameter space. (f) Out‐of‐sample blind prediction using Leave‐One‐Out Cross‐Validation (LOOCV).

Rather than fitting each specimen independently, we performed a joint inversion across all eight groups of dimensionless experimental data. Under unified physical constraints, the calibrated global parameter set (Table S3) achieves *R* ^2^ > 0.997 for every individual dataset Figure S6.

Because the joint inversion operates in the dimensionless domain, its output must be mapped back to physical units. This inverse mapping requires two boundary anchors for any given pre‐strain: the residual resistance (Δ*R*/*R*_0_|_ε = 0_) and the steady‐state peak resistance ($\Delta R/{R_{0}|}_{\varepsilon =\varepsilon_{\mathrm{pre}}}$). For the residual resistance, the eight measured values — together with the physical origin (zero pre‐strain yields zero residual resistance) — were interpolated using a shape‐preserving piecewise cubic Hermite polynomial (PCHIP) to construct a continuous function.

For the steady‐state peak resistance, the mapping exploits a self‐consistent relationship within the experimental data. Figure 3d,e show that the first‐cycle loading curves across all pre‐strain levels collapse onto a single master envelope (*R* ^2^ > 0.99), and the initial loading peaks lie along this envelope. Because the steady‐state response closely follows the first‐cycle unloading profile (Pearson r > 0.999, Figure S4) with only a systematic amplitude offset, the steady‐state peaks must trace a curve geometrically congruent to the master loading envelope. We therefore applied the loading‐phase electromechanical Equation (3) directly to fit the steady‐state peak resistance as a function of pre‐strain (Figure 3i, red markers and dashed line), obtaining *R* ^2^ = 0.9996 (Table S4). With both anchors established, a linear rescaling of the dimensionless output reconstructs the complete electromechanical curve in physical space for any prescribed pre‐strain configuration, from which a continuous response surface can be generated. The complete workflow is illustrated in Figure 5a–d. Model predictions for all eight pre‐stretch levels are compared with the experimental data in Figure S7; two representative cases — 15 and 3 mm pre‐stretch — are highlighted in Figure 5e. The model exhibits strong predictive accuracy: even for the 3 mm group, which yields the lowest fitting precision among all specimens, *R* ^2^ reaches 0.9896.

To evaluate the model's ability to generalize to unseen pre‐strain values, we performed Leave‐One‐Out Cross‐Validation (LOOCV). In each round, the dataset of one specimen was withheld; the 12‐parameter joint calibration, PCHIP interpolation, and peak‐resistance fitting were reconstructed from the remaining seven groups, and a fully blind prediction was generated for the withheld specimen. For all interior design‐space configurations (5–30 mm pre‐stretch), the blind predictions achieve *R* ^2^ > 0.9701 in physical space Figure S8. The 3 mm pre‐stretch specimen yields a lower *R* ^2^ (0.8164), attributable to one‐sided interpolation at the design‐space edge; nevertheless, the predicted curve shape tracks the experimental trend, with the discrepancy confined to a peak‐resistance offset Figure 5f.

To verify that this generalization accuracy stems from the physics‐based model structure rather than from the density of calibration data alone, we repeated the LOOCV using an optimized radial‐basis‐function (RBF) response surface constructed from the same eight datasets (Note S4). The two approaches achieve comparable accuracy at interior points (mean *R* ^2^ = 0.989 vs. 0.992), but at the 3 mm boundary the unconstrained RBF collapses to *R* ^2^ = −0.46 while the physics‐constrained model retains *R* ^2^ = 0.82 — confirming that the governing equations provide predictive content beyond what interpolation of the calibration data can deliver.

This capability marks a qualitative shift in the microcrack sensor field: existing electromechanical models must be recalibrated for each specimen and can only interpret conditions already tested, whereas the globally calibrated model generates the complete response at any untested pre‐strain within the design space, providing the quantitative foundation for the inverse‐design optimization developed in the following section.

## 4 Inverse Design of Pre‐Strain and Experimental Validation

### 4.1 Construction of the Multi‐Dimensional Response‐Surface Database

Searching for the globally optimal pre‐strain within a continuous design space requires the complete strain–resistance response at arbitrary pre‐strain configurations — a dataset that exhaustive experimental testing cannot practically deliver. As established in Section 3.6, however, the electromechanical model possesses predictive capability: the electromechanical behavior of any pre‐strained specimen is deterministically confined to the analytical response surface Figure 5d, provided that the working strain does not exceed the pre‐strain value. This predictive power enables the systematic construction of a high‐density design database directly from the electromechanical model, without additional experimentation.

As depicted in Figure 6a,b, within the range of 3–30 mm, the pre‐stretch was discretized at intervals of 0.1 mm, yielding a total of 271 pre‐stretch configurations. For each pre‐stretch level, the working strain was incrementally increased from 1% in steps of 1% up to the maximum working strain permitted by that configuration, thereby exhaustively traversing all feasible working ranges and mapping the corresponding response characteristics. For each (pre‐stretch, working range) combination, the analytical model was used to uniformly generate 1000 data points within the working range, from which the following three performance metrics were extracted: **Sensitivity** was quantified as the secant slope of the response curve across the operating window [0, ε_work_].

**FIGURE 6:**
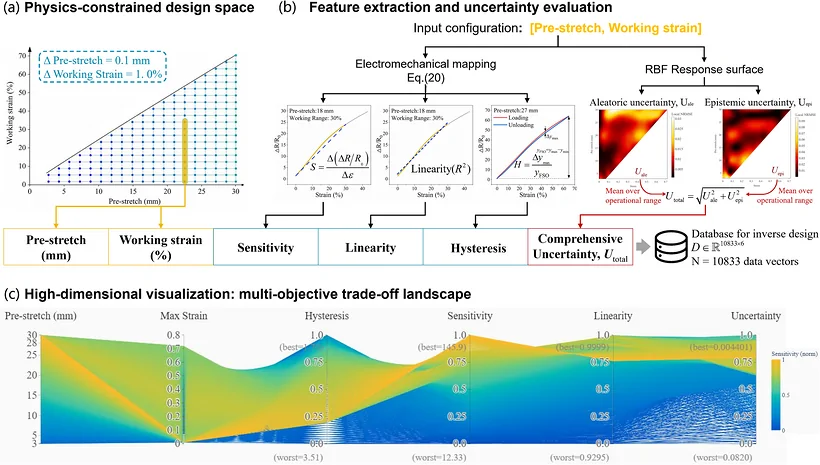
Construction and visualization of a physics‐constrained multi‐objective sensor design database. (a) Design space defined by pre‐stretch displacement and working strain. (b) Feature‐extraction pipeline for sensitivity, linearity, hysteresis, and total uncertainty. (c) Parallel‐coordinate plot showing multi‐objective trade‐offs across 10 833 design vectors.

**Linearity** was characterized by the coefficient of determination (*R* ^2^) from a linear fit to the response within the operating window.

**Hysteresis** was evaluated via the maximum‐difference method. As illustrated in Figure 3l, hysteresis remains approximately invariant across different working sub‐ranges at a given pre‐strain, reflecting an intrinsic property of the crack network topology established at that pre‐strain level. Accordingly, hysteresis was computed from the full loading–unloading loop at the maximum permissible working strain rather than from truncated sub‐intervals. The loading and unloading response surfaces were generated independently from the analytical model and concatenated to construct a composite cyclic response surface Figure S9. This approach ensures globally consistent hysteresis assessment for a given physical design and eliminates measurement artifacts arising from sub‐range truncation.

**Uncertainty quantification**. Favorable model‐predicted performance values alone do not guarantee engineering reliability: a design point may reside in a data‐sparse region where epistemic confidence is inherently low, or coincide with conditions of poor experimental reproducibility. Assessing design configurations by sensitivity, linearity, and hysteresis alone implicitly treats both model predictions and experimental observations as deterministic — an assumption that must be relaxed. We therefore posit that every extracted performance metric embeds two physically distinct sources of uncertainty. Epistemic uncertainty (*U*_epi_) captures the approximation error of the analytical model relative to the true physical process. Aleatoric uncertainty (*U*_ale_) reflects the irreducible stochasticity arising from viscoelastic material behavior, inter‐specimen microstructural variability, and measurement noise; it is characterized by the local standard deviation of the mean steady‐state response at each strain level (error bars in Figure 3i). The two contributions are statistically independent and are combined into a total uncertainty by root‐sum‐of‐squares composition: $U_{\mathrm{total}}=\sqrt{U_{\mathrm{epi}}^{2}+U_{\mathrm{ale}}^{2}}$. To quantify both fields across the continuous design space, the LOOCV residuals at the eight calibration points are converted into spatially resolved local NRMSE profiles and mapped via thin‐plate‐spline RBF interpolation, producing an epistemic uncertainty field *U*_epi_ that assigns progressively higher penalty to data‐sparse regions; the aleatoric uncertainty field *U*_ale_ is constructed by the same procedure using the experimental cycle‐to‐cycle variability as input (Note S5). This composite uncertainty serves as a penalty term and is jointly incorporated with sensitivity, linearity, and hysteresis into the subsequent multi‐objective optimization framework, explicitly precluding design points that are mathematically optimal yet physically unreliable — whether the unreliability originates from insufficient model fidelity or poor experimental reproducibility.

Exhaustive traversal of all 271 pre‐stretch configurations and their associated operating windows yields a database of 10 833 records, each comprising pre‐strain, working range, hysteresis, sensitivity, linearity, and composite uncertainty. The complete construction pipeline is depicted in Figure 6a,b. To elucidate the nonlinear coupling between design parameters and multidimensional sensing performance, a parallel coordinate plot (PCP) is employed for high‐dimensional visualization Figure 6c, with all metrics mapped to [0, 1] via min–max normalization.

### 4.2 Inverse Design for Smart‐Tire Strain Monitoring

To validate the engineering applicability of the established database, we demonstrate an inverse design framework for pre‐strain optimization, targeting smart‐tire strain sensors. Under typical passenger vehicle operating conditions, the strain amplitude of the tire inner liner generally ranges from 5% to 6% [44]. To ensure a sufficient safety margin against extreme impact loads, the target dynamic range of the sensor was rigorously defined as 10% strain.

The multi‐objective decision‐making framework for this inverse design is illustrated in Figure 7a. Initially, 259 candidate pre‐stretch configurations satisfying the 10% strain prerequisite were extracted from the database. Subsequently, multi‐objective optimization was performed within a four‐dimensional parameter space—comprising sensitivity, linearity, hysteresis, and composite uncertainty—yielding 216 Pareto‐optimal solutions. To further refine the feasible region, stringent engineering constraints were applied: degraded configurations with linearity *R* ^2^ < 0.98, hysteresis *H* > 2%, and composite uncertainty *U*_total_ > 0.05 were discarded, retaining 97 rigorously feasible Pareto solutions. The robustness of this result was verified by varying the uncertainty threshold from 0.03 to 0.10: the knee‐point solution and the feasible Pareto solution set are both completely invariant across the entire range (Table S7), confirming that the recommended design is governed by the four‐objective Pareto‐front geometry rather than by the specific threshold value.

**FIGURE 7:**
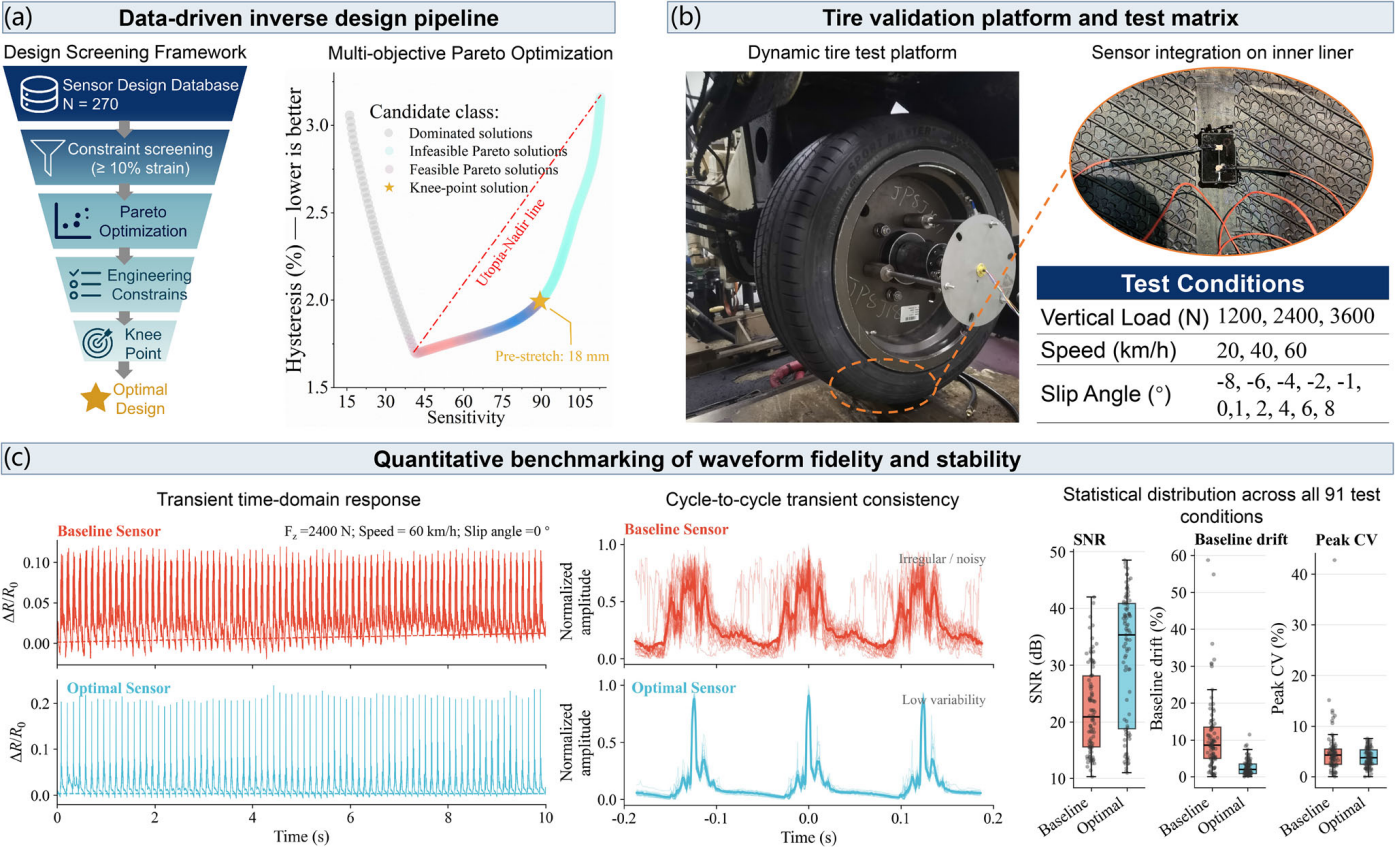
Inverse design and dynamic tire‐platform validation of the optimized sensor. (a) Sequential screening framework and Pareto optimization for sensor inverse design. (b) Sensor integration on the tire inner liner and test matrix covering vertical load, speed, and slip angle. (c) Benchmarking of the baseline and optimized sensors using time‐domain responses, cycle‐to‐cycle consistency, and statistical distributions of SNR, baseline drift, and peak coefficient of variation (CV) across 91 valid test conditions.

Because inherent trade‐offs exist among the performance metrics within this multidimensional space, we introduced the knee‐point criterion to identify the global optimal solution. On the feasible Pareto front, the knee point is mathematically defined as the solution exhibiting the maximum orthogonal projection distance to the line connecting the Utopia point (the theoretical optimum for all objectives) and the Nadir point (the acceptable worst‐case boundaries). This criterion enables the objective identification of a design that balances multidimensional marginal benefits, circumventing the bias of subjective weight allocation. Ultimately, the algorithm identified a global recommended configuration of 18 mm pre‐stretch, corresponding to an equivalent pre‐strain of 43.0%. To contextualize this recommendation, Table S5 compares the model‐predicted performance metrics at 10% working strain for six representative pre‐stretch levels spanning the design space, including the 10, 20, and 30 mm configurations. Sensitivity increases monotonically with pre‐stretch, while hysteresis likewise rises; the two objectives are in direct competition. The 18 mm knee point is the highest‐sensitivity configuration that simultaneously satisfies all engineering constraints (*R* ^2^ ≥ 0.98, *H* ≤ 2%, *U*_total_ ≤ 0.05) — configurations at 20 mm and above exceed the hysteresis bound Figure S12.

### 4.3 Smart‐Tire Bench Validation

In intelligent‐tire systems, the inner‐liner strain signal serves as the primary information carrier for tire‐state perception. Each tire revolution generates a characteristic waveform whose transient features mark the entry into and exit from the contact patch. Downstream estimation schemes exploit this per‐revolution information — whether through physics‐based features such as contact‐patch length, or through data‐driven models operating directly on the raw waveform — to infer the vertical load, slip state, and road‐surface condition [15, 16, 19, 20, 21]. These tire‐state estimates in turn constitute the sensing input for chassis‐control functions such as semi‐active suspension control, anti‐lock braking, and friction‐adaptive stability control [45, 46, 47]. The bench validation below therefore serves a 2‐fold purpose: to verify that the inversely designed sensor sustains this information‐carrying role under realistic rolling conditions, and to quantify the signal attributes — noise level, baseline stability, and cycle‐to‐cycle consistency — on which downstream feature extraction directly depends.

To this end, the optimal configuration (43.0% pre‐strain) and an untreated baseline sensor were co‐mounted along the crown centerline of the same tire inner liner Figure 7b. A nominal matrix of 99 operating conditions was planned; 91 were retained after excluding conditions with unstable tire‐rig operation or incomplete signal acquisition. Vertical load, rolling speed, and slip angle were swept on an MTS flat‐trac tire force‐and‐moment test rig Figure S10, with both sensors acquiring data synchronously in each condition to ensure pairwise comparability.

Figure 7c compares the time‐domain responses of the two sensors under a representative condition (F_z​_ = 2400 N, speed = 60 km h^−^^1^, slip angle = 0°). The baseline sensor exhibits pronounced low‐frequency baseline drift and broadband noise, with substantial stochastic fluctuations in the per‐revolution transient peaks. In contrast, the inversely designed sensor delivers a clean waveform with a stable baseline and sharp transient features. When 20 consecutive contact‐patch waveforms are phase‐aligned to their peak values (Figure 7c, middle), the baseline sensor's trace envelope diverges markedly, whereas the inversely designed sensor's waveforms overlap closely, indicating superior cycle‐to‐cycle repeatability.

To move beyond single‐condition observations, a Wilcoxon signed‐rank test was applied across all 91 conditions to evaluate signal quality (SNR), baseline stability (drift rate), and cycle‐to‐cycle consistency (peak CV) — the three attributes on which downstream tire‐state estimation directly depends (Figure 7c, right). The median signal‐to‐noise ratio (SNR) of the inversely designed sensor reaches 35.31 dB, up from 20.91 dB for the baseline (a 68.8% improvement; *p* = 3.11 × 10^−11^). Separating the signal and noise contributions shows that this +14.4 dB gain is dominated by a 75.6% reduction in the noise floor (+12.2 dB, *p* = 6.73 × 10^−^^5^), with signal amplification accounting for only +1.4 dB (×1.17, *p* = 0.067, not significant). In approximately 25% of conditions — predominantly at high vertical loads and large slip angles — the noise levels of the two sensors are comparable, as the mechanical excitation itself dominates the noise floor Figure S11. The median baseline drift rate decreases from 8.52% to 1.93% (a 77.3% reduction; *p* = 1.19 × 10^−15^). The peak CV, however, shows only a modest difference — 4.26% vs. 3.73% — that does not reach significance (*p* = 0.109). This pattern carries a clear physical interpretation: the dominant degradation mode of the baseline sensor under dynamic cycling is not peak‐capture instability but baseline drift and noise contamination driven by irreversible microstructural evolution of the conductive network. Pre‐straining drives the crack network to a stable topological state prior to deployment, eliminating this mechanism at its source.

The bench‐test results confirm that the inversely designed sensor (43.0% pre‐strain) preserves peak‐capture stability while resolving the baseline drift and low‐SNR challenges that plague flexible sensors under high‐frequency dynamic cycling, experimentally validating the effectiveness of the physics‐driven inverse‐design framework in engineering applications. For the tire‐state‐estimation functions outlined above, these gains translate directly: the stabilized baseline preserves the amplitude reference on which load inference relies, the elevated SNR sharpens the contact‐patch signatures that anchor feature extraction, and the improved cycle‐to‐cycle repeatability reduces the variance of per‐revolution estimates — easing the burden on downstream signal conditioning and estimation algorithms.

Table 2 situates the inversely designed sensor among representative crack‐based and other state‐of‐the‐art flexible strain sensors. No reported sensor dominates across all four metrics: the highest gauge factors are attained at the expense of linearity or working range, whereas the present device — with the highest linearity (*R* ^2^ = 0.9994) and the lowest characterized hysteresis (1.99%) at a moderate gauge factor of 89.3 — occupies the balanced operating point that the 10% working‐strain application demands. More fundamentally, this operating point is not the fortunate outcome of discrete trial‐and‐error but the knee‐point solution of a continuous, uncertainty‐penalized four‐objective optimization; the same framework can relocate the operating point for any other prescribed requirement.

**TABLE 2:** Performance comparison of the inversely designed sensor with representative flexible strain sensors reported in the literature.

Ref.	Sensing mechanism	GF (at 10% strain unless noted)	Linearity *R* ^2^ (at 10% strain)	Hysteresis	Working range
[31]	Pt film, channel crack	2000 (at 2%)	0.83^†^ (at 2%)	n.r.	2%
[39]	Au film, channel crack	3721^†^ (at 1%)	0.4629^†^ (at 1%)	n.r.	1%
[34]	Pt/SACNT bilayer crack	3930^†^	0.996^†^	n.r.	100%
[36]	CNF‐reinforced crack	125^†^	0.83^†^	n.r.	130%
[33]	micro‐groove crack	1471^†^	0.4257^†^	n.r.	6%
[29]	network–channel boundary crack	562.6^†^	0.993^†^	n.r.	120%
[28]	fatigue crack	1971^†^	0.82^†^	11.3%^†^	3%
[48]	Gated Liquid Metal Channel	7.65^†^	0.9^†^	n.r.	140%
[49]	Liquid metal with foam	4.5^†^	0.9481^†^	n.r.	120%
This work	Pre‐strained microcrack	89.3	0.9994	1.99%	43%

^†^ Digitized from published figures; n.r. = not reported.

Although the present validation confirms short‐to‐medium‐term dynamic performance, the actual tire service environment imposes additional challenges not captured in this study: prolonged cyclic bending over millions of revolutions, temperature excursions from ambient to above 80°C, progressive adhesion degradation at the sensor–rubber interface, and abrasive wear of the tire inner liner. Whether the pre‐strain‐stabilized crack topology retains its electrical integrity under such cumulative loading remains an open question. Long‐term durability assessment under service‐representative thermo‐mechanical cycling is therefore identified as a critical next step toward engineering deployment. Similarly, a multi‐level tire‐platform comparison benchmarking the knee‐point design against non‐optimal pre‐stretch controls is warranted to experimentally verify the predicted trade‐off advantages (Table S7).

## 5 Conclusion

This work addresses a long‐standing challenge in the flexible piezoresistive sensor field: how to determine the optimal pre‐strain magnitude for microcracked conductive layers when sensitivity, linearity, hysteresis, and working range are inherently coupled through competing trade‐offs.

(1) An analytical electromechanical model grounded in microcrack evolution mechanics was developed. The model captures two physically distinct regimes — progressive fragmentation of the conductive network during initial loading, and reversible opening–closure of pre‐existing cracks during steady‐state cycling — within a unified mathematical framework. Independent fitting yields *R* ^2^ ≥ 0.997 across all eight pre‐strain levels for both the first‐cycle and steady‐state responses.

(2) Through physically motivated parameter classification, the model was extended from a per‐specimen curve‐fitting tool to a globally calibrated predictive framework. Nine intrinsic material constants and a single pre‐strain‐dependent state variable (*k_s_* ​, parameterized as an exponential decay function) — twelve parameters in total — were jointly calibrated against all eight datasets in dimensionless space. Leave‐one‐out cross‐validation confirms blind‐prediction *R* ^2^ > 0.97 for all interior design‐space configurations (5–30 mm pre‐stretch), establishing predictive capability for untested configurations within the calibrated design space. To the best of our knowledge, this is the first electromechanical model in the microcrack‐based flexible sensor literature that predicts — rather than merely interprets — the complete response at an arbitrary, untested pre‐strain configuration. Notably, the physics‐constrained model structure reduces the calibration requirement to only eight pre‐strain levels — substantially fewer than the hundreds or thousands of training samples typically required by data‐driven surrogate models — while maintaining predictive fidelity across the continuous design space.

(3) An inverse‐design framework was constructed by coupling the predictive model with a composite uncertainty penalty that fuses epistemic (LOOCV‐derived) and aleatoric (cycle‐to‐cycle variability) contributions, together with multi‐objective Pareto optimization using a knee‐point selection criterion. For a target working range of 10% strain (smart‐tire application), the framework identifies 18 mm pre‐stretch (43.0% pre‐strain) as the globally recommended configuration without subjective weight assignment.

(4) Bench tests on an MTS flat‐trac rig across 91 operating conditions validate the inversely designed sensor against an untreated baseline. The median SNR improves from 20.91 to 35.31 dB (*p* = 3.11 × 10^−^^1^ ^1^), and the median baseline drift rate decreases from 8.52% to 1.93% (*p* = 1.19 × 10^−^^1^ ^5^), while peak‐capture stability is preserved.

Taken together, this work demonstrates a paradigm shift in flexible sensor development — from empirical trial‐and‐error forward design, in which pre‐strain is selected by fabricating and testing discrete specimens, to demand‐driven inverse design, in which the target operating condition dictates the optimal pre‐strain through a physics‐based analytical pipeline. The methodology is not restricted to piezoresistive sensors or pre‐strain engineering; the global‐calibration‐to‐inverse‐design framework is applicable, in principle, to any microcrack‐based functional system in which a structural design parameter governs multi‐dimensional performance trade‐offs.

## Author Contributions

**Jia‐Chen Shang**: conceptualization, methodology, formal analysis, writing – original draft, writing – review and editing. **Rui Wang**: conceptualization, data curation, investigation, visualization. **Bo Lu**: formal analysis, resources. **Ming‐Liang Song**: data curation, software, validation. **Yu‐Yi Chen**: investigation, methodology, supervision. **Kuo Han**: validation, visualization. **Xin‐Jie Feng**: software, visualization. **Bin Sun**: formal analysis, data curation. **Yao‐Guang Cao**: formal analysis, methodology, supervision. **Xiao‐Yu Yan**: investigation, software, resources. **Shi‐Chun Yang**: methodology, writing – review and editing, project administration, funding acquisition.

## Funding

This work was supported by the National Natural Science Foundation of China (Grant No. U22A2042).

## Conflicts of Interest

The authors declare no conflicts of interest.

## Supporting information




**Supporting File**: advs77146‐sup‐0001‐SuppMat.docx.

## Data Availability

The data that support the findings of this study are available from the corresponding author upon reasonable request.
