# Supplementary material for: Inverse Design of Pre‐Strain via a Predictive Electromechanical Model for Enhanced Strain Sensing
Source: Adv Sci (Weinh). 2026 Aug 29:e77146. Online ahead of print. doi: 10.1002/advs.77146 (PMC13525424; doi:10.1002/advs.77146)
Supplement: Supplementary file 1 [file ADVS-9999-e77146-s001.docx]

Supplementary Material for

**Inverse Design of Pre-Strain via a Predictive Electromechanical Model for Enhanced Strain Sensing**

Jia-Chen Shang, Rui Wang, Bo Lu, Ming-Liang Song, Yu-Yi Chen, Kuo Han, Xin-Jie Feng, Bin Sun, Yao-Guang Cao, Xiao-Yu Yan*, and Shi-Chun Yang*

J. C. Shang, R. Wang, K. Han, X. J. Feng, B. Sun, S. C. Yang*

School of Transportation Science and Engineering, Beihang University, 37 Xueyuan Road, Haidian District, Beijing 100191, PR China

E-mail: yangshichun@buaa.edu.cn

Bo Lu, Ming-Liang Song

Shandong Linglong Tyre Co., Ltd., 777 Jinlong Road, Zhaoyuan, Shandong 265400, PR China

Y. Y. Chen, Y. G. Cao

Hangzhou International Innovation Institute, Beihang University, Hangzhou, PR China

Y. G. Cao

State Key Lab of Intelligent Transportation System, Beijing, PR China

X. Y. Yan*

Research Institute of Aero-Engine, Beihang University, 37 Xueyuan Road, Haidian District, Beijing 100191, PR China

E-mail: yanxiaoyu@buaa.edu.cn

S. C. Yang*

Innovation Center of New Energy Vehicle Digital Supervision Technology and Application for State Market Regulation, Beijing 100191, PR China

**S1. Supplementary figures.**

**Figure S1**. Nominal strain in the gauge section of the Type 2 dumbbell specimen as a function of crosshead displacement, showing a linear relationship up to fracture at 34.2 mm.

**Figure S2**. Pearson correlation heat map of normalized unloading curves

**Figure S3**. Per-group comparison between the experimental first-cycle electromechanical responses and the analytical model (Equations (3) and (6)) for all eight pre-stretch levels. Coefficients of determination are indicated in each panel; the fitted parameters are listed in Table S1.

**Figure S4**. Pearson correlation coefficients between mean steady-state response and first-cycle unloading curve

**Figure S5**. Per-group comparison between the experimental mean steady-state response and the analytical model (Equations (6)) for all eight pre-stretch levels. Coefficients of determination are indicated in each panel; the fitted parameters are listed in Table S2.

**Figure S6**. Joint inversion fitting results for all eight datasets in dimensionless space

**Figure S7**. Inverse-mapped complete electromechanical hysteresis curves in physical space

**Figure S8.**  LOOCV blind prediction results for all eight pre-stretch levels

**Figure S9**. Composite cyclic response surface from concatenated loading/unloading surfaces

**Figure S10**. All 91 operating conditions on an MTS flat-trac tire force-and-moment test rig

**Figure S11**. Decoupling of signal and noise across all 91 test conditions

**Figure S12.** Trade-off between sensitivity and hysteresis at 10% working strain for six representative pre-stretch configurations (Table S5). The dashed line indicates the engineering constraint *H* = 2%. The 18 mm knee-point solution (star) is the highest-sensitivity configuration within the feasible region; all configurations at 20 mm and above violate the hysteresis constraint.

**S2. Supplementary tables.**

**Table S1**. Fitted analytical parameters characterizing the initial electromechanical response cycle.

| **Pre-stretch** | ***R*²** | $l_{\text{c}}^{\text{sat}}$**(mm)**  | $k_{\text{c1}}$ | $k_{\text{c2}}$ | $N_{\text{sat}}$ | $k_{\text{n}}$ | $\lambda$ | $\alpha$ | $\beta$ | $R_{\text{c}}^{\left(0\right)}$(Ω)  | $k_{\text{s}}$ |
| --- | --- | --- | --- | --- | --- | --- | --- | --- | --- | --- | --- |
| 3 mm | **0.9997** | 2 | 1.0001 | 12.3348 | 10.0682 | 8.0718 | 0.1000 | 1.7908 | 0.7430 | 3046.3623 | 65.6001 |
| 5 mm | **1.0000** | 2 | 1.0000 | 5.7547 | 14.6318 | 8.8680 | 0.1000 | 4.1411 | 0.8068 | 1815.0750 | 48.2276 |
| 10 mm | **0.9999** | 2 | 2.2700 | 10.8248 | 5.0000 | 17.7497 | 1.1313 | 3.3262 | 0.7398 | 2207.2190 | 28.9630 |
| 15 mm | **0.9998** | 2 | 2.3903 | 8.4339 | 6.2021 | 15.7092 | 4.2447 | 4.1613 | 0.6799 | 1652.4512 | 23.9097 |
| 20 mm | **0.9997** | 2 | 3.0000 | 5.0647 | 12.0460 | 11.2853 | 2.8568 | 7.2044 | 0.7813 | 1441.3067 | 20.3569 |
| 25 mm | **0.9999** | 2 | 2.3909 | 4.2027 | 11.3802 | 11.2248 | 4.6587 | 9.4076 | 0.8386 | 1523.4324 | 17.6087 |
| 28 mm | **0.9998** | 2 | 2.1810 | 5.3067 | 8.1986 | 9.6188 | 5.0000 | 10.0000 | 0.8591 | 1111.2457 | 18.3417 |
| 30 mm | **0.9998** | 2 | 2.4631 | 4.3909 | 10.6217 | 9.4885 | 4.2639 | 10.0000 | 0.8049 | 1157.8944 | 16.3407 |

**Table S2.** Fitted analytical parameters for the mean steady-state response.

| **Pre-stretch** | ***R*²** | $l_{\text{c}}^{\text{sat}}$**(mm)**  | $k_{\text{c1}}$ | $k_{\text{c2}}$ | $N_{\text{sat}}$ | $k_{\text{n}}$ | $\lambda$ | $\alpha$ | $\beta$ | $R_{\text{c}}^{\left(0\right)}$(Ω)  | $k_{\text{s}}$ |
| --- | --- | --- | --- | --- | --- | --- | --- | --- | --- | --- | --- |
| 3 mm | **0.9998** | 2 | 1.1412 | 8.4733 | 15.6071 | 7.2016 | 6.7337 | 0.5000 | 1.0000 | 2116.7869 | 83.0082 |
| 5 mm | **0.9999** | 2 | 2.9032 | 3.0528 | 40.0472 | 5.1902 | 0.0500 | 6.9049 | 0.8874 | 964.2270 | 51.3654 |
| 10 mm | **0.9999** | 2 | 3.0000 | 3.0000 | 26.1498 | 4.2350 | 0.0500 | 13.4282 | 0.6894 | 1579.4175 | 28.2943 |
| 15 mm | **0.9998** | 2 | 3.0000 | 3.0000 | 17.2807 | 3.3070 | 0.0500 | 23.8292 | 0.6474 | 1835.0318 | 21.8231 |
| 20 mm | **0.9997** | 2 | 1.3444 | 3.8626 | 5.0000 | 3.0000 | 2.5984 | 25.0000 | 0.6817 | 3961.5056 | 23.9284 |
| 25 mm | **0.9998** | 2 | 1.1958 | 4.8947 | 5.0000 | 3.0000 | 10.0000 | 25.0000 | 0.7890 | 3532.7491 | 19.7735 |
| 28 mm | **0.9997** | 2 | 1.4384 | 5.3923 | 5.0000 | 3.0000 | 0.0500 | 25.0000 | 0.8296 | 2338.5101 | 18.8402 |
| 30 mm | **0.9997** | 2 | 1.4474 | 5.6270 | 5.0000 | 3.0000 | 0.0500 | 25.0000 | 0.7919 | 2447.9580 | 17.0023 |

**Table S3.** Globally calibrated parameter set obtained via joint inversion across all eight pre-stretch levels in dimensionless space.

| **Pre-stretch** | ***R*²** | $l_{\text{c}}^{\text{sat}}$ | $k_{\text{c1}}$ | $k_{\text{c2}}$ | $N_{\text{sat}}$ | $k_{\text{n}}$ | $\lambda$ | $\alpha$ | $\beta$ | $R_{\text{c}}^{\left(0\right)}$ | A | B | C | $k_{\text{s}}$ |
| --- | --- | --- | --- | --- | --- | --- | --- | --- | --- | --- | --- | --- | --- | --- |
| 3 mm | **0.9972** | 2.0000 | 0.2000 | 2.0000 | 126.6658 | 0.1722 | 0.5000 | 3.0000 | 0.7248 | 898.5087 | 10.3825 | 0.0768 | 3.7627 | 12.0087 |
| 5 mm | **0.9980** |  |  |  |  |  |  |  |  |  |  |  |  | 10.8345 |
| 10 mm | **0.9984** |  |  |  |  |  |  |  |  |  |  |  |  | 8.5795 |
| 15 mm | **0.9991** |  |  |  |  |  |  |  |  |  |  |  |  | 7.0436 |
| 20 mm | **0.9975** |  |  |  |  |  |  |  |  |  |  |  |  | 5.9974 |
| 25 mm | **0.9983** |  |  |  |  |  |  |  |  |  |  |  |  | 5.2848 |
| 28 mm | **0.9992** |  |  |  |  |  |  |  |  |  |  |  |  | 4.9716 |
| 30 mm | **0.9988** |  |  |  |  |  |  |  |  |  |  |  |  | 4.7995 |

**Table S4.** Fitted parameters for the steady-state peak resistance using Equation (3).

| ***R*²** | $l_{\text{c}}^{\text{sat}}$ | $k_{\text{c1}}$ | $k_{\text{c2}}$ | $N_{\text{sat}}$ | $k_{\text{n}}$ | $\lambda$ | $\alpha$ | $\beta$ |
| --- | --- | --- | --- | --- | --- | --- | --- | --- |
| **0.9990** | 2.0000 | 3.0000 | 4.2110 | 20.0723 | 17.7171 | 3.8275 | 3.6239 | 0.5415 |

**Table S5.** Model-predicted performance metrics at 10% working strain for six representative pre-stretch configurations. The 18 mm knee-point solution (bold) is identified by the Pareto optimization of Section 4.2. The engineering constraint boundary for hysteresis is *H* = 2%.

| **Pre-stretch (mm)** | **Sensitivity** | **Linearity *R*²** | **Hysteresis (%)** | ***U*_total_** |
| --- | --- | --- | --- | --- |
| 10 | 50.7 | 0.9946 | 1.73 | 0.026 |
| 15 | 80.5 | 0.9998 | 1.88 | 0.013 |
| **18 (knee point)** | **89.3** | **0.9994** | **1.99** | **0.023** |
| 20 | 93.5 | 0.9994 | 2.09 | 0.029 |
| 25 | 102.1 | 0.9995 | 2.48 | 0.011 |
| 30 | 112.4 | 0.9997 | 3.16 | 0.026 |

**Note S3. Complete Derivation of the Microcrack-Based Electromechanical Model**

This note provides the step-by-step derivation of the governing equations presented in Section 3.4 of the main text. The geometric abstraction and equivalent-circuit topology follow Figure 4 therein: the cracked conductive layer (length *L*, width *W*, thickness *h*) is idealized as a periodic array of *n* parallel cracks of length *l*_c_, width *w*_c_, and spacing *d* = *L*/(*n*+1); the minimum repeating unit comprises the bridge resistance *R*_1_ parallel to the cracks, the island resistance *R*_2_ perpendicular to them, and the crack-interface contact resistance *R*_c_.

**S3.1 Loading phase: fragmentation of the conductive network**

In situ SEM observation (Figure 4(b-i), main text) shows that newly formed crack faces separate abruptly without bridging contact; the crack interface is therefore an open circuit during initial loading, and the unit-cell resistance is the series combination of the bridge and island contributions.

|  | $R_{\text{u}}^{\left(1\right)}=R_{1}+R_{2}$ | (Equation (2), main text) |
| --- | --- | --- |

The resistance *R*_1_ of the narrow strip parallel to the crack direction (the "bridge" ^[1, 2]^) is expressed as:

$R_{1}=\frac{\rho_{1}l_{\text{c}}}{dh}$ (S1)

where h is the conductive-layer thickness (h = 0.03 mm ^[3]^) and $\rho_{1}$ is the resistivity of the conductive layer in the width direction, with an unloaded value of $\rho_{1}^{\left(0\right)}=$ 17.751 Ω·mm. Its relationship with the nominal strain in the length direction is:

$\rho_{1}=\rho_{1}^{\left(0\right)}\left(\lambda\varepsilon+1\right)$ (S2)

where $\lambda$ is a coefficient characterizing the degree of influence of tensile strain on the width-direction resistivity.

The resistance $R_{2}$ of the portion perpendicular to the crack direction connecting the two side strips (the "islands" ^[1, 2]^) is expressed as:

$R_{2}=\frac{\rho_{2}\left(d+w_{\text{c}}\right)}{\left(l-l_{\text{c}}\right)h}$ (S3)

where the crack width $w_{\text{c}}$ scales linearly with strain as ( = 0.022 mm), based on the experimental results in Figure 2(d) of the main text. $\rho_{2}$ is the resistivity of the conductive layer in the length direction, with an unloaded value of $\rho_{2}^{\left(0\right)}=12.367$ Ω·mm, ^[3]^ and its strain dependence satisfies:

$\rho_{2}=\left(\alpha\varepsilon^{\beta}+1\right)\rho_{2}^{\left(0\right)}$ (S4)

Based on the crack-length evolution characteristics in Figure 2(c) of the main text, the crack length during initial loading is fitted by a Gompertz function:

$l_{\text{c}}^{(1)}=l_{\text{c}}^{\text{sat}}\cdot\left[\exp\left[-k_{\text{c1}}\cdot\exp(-k_{\text{c2}}\cdot\varepsilon)\right]-\exp\left(-k_{\text{c1}}\right)\right]$ (S5)

where $l_{\text{c}}^{\text{sat}}$ is the saturation crack length, whose maximum value equals the unit-cell dimension along the crack-length direction *l*, which in turn equals the sensing-layer width *W* (i.e., *l* = *W*); *k*_c1_ and *k*_c2_ are coefficients governing the crack-length propagation rate.

The entire conductive layer constitutes a network of $R_{\text{u}}$ units, and the total resistance *R* of the sensing layer is:

$R=\left(n+1\right)R_{\text{u}}^{(1)}$ (S6)

In the initial unloaded state, no cracks exist in the conductive layer (*n* = 0), and the initial resistance *R*_0_ is:

$R_{0}=\frac{\rho_{2}^{\left(0\right)}L}{lh}$ (S7)

Substituting Equations (S1)–(S7), together with the crack-density saturation law (Equation (1), main text), into the definition of Δ*R*/*R*_0_ yields the loading-phase governing equation,

|  |  | (Equation (3), main text) |
| --- | --- | --- |

where $\Gamma=\frac{\rho_{1}^{\left(0\right)}}{\rho_{2}^{\left(0\right)}}$ is the ratio of the initial conductivities in the two directions,, and $\Phi(\varepsilon)=\alpha\varepsilon^{\beta}+1$.

**S3.2 Unloading phase: reversible crack closure**

During unloading, the crack topology formed on loading is frozen: the maximum crack length and the crack spacing no longer change, as corroborated by the constant crack spacing in Figure 2(e) of the main text. The bridge geometry is accordingly fixed, and its resistance $R_{1}$ is expressed as:

$R_{1}^{\left(2\right)}=\frac{\rho_{1}l_{\text{c}}^{\max}}{d^{\text{fix}}h}$ (S8)

where $l_{\text{c}}^{\max}$ is the maximum crack length reached during loading and $d^{\text{fix}}$ is the crack spacing at maximum strain:

$\begin{matrix} l_{\text{c}}^{\max}=l_{\text{c}}^{\text{sat}}\cdot\left[\exp\left[-k_{\text{c1}}\cdot\exp(-k_{\text{c2}}\cdot\varepsilon_{\max})\right]-\exp\left(-k_{\text{c1}}\right)\right] \\ d^{\text{fix}}=L/\left[N_{sat}[1-\exp(-k_{\text{n}}\cdot\varepsilon_{\max})]+1\right] \end{matrix}$ (S9)

Because the crack width $w_{\text{c}}$ varies with strain, the island geometry changes accordingly, and its resistance $R_{2}$ is expressed as:

$R_{2}^{\left(2\right)}=\frac{\rho_{2}\left(d^{\text{fix}}+w_{\text{c}}\right)}{\left(l-l_{\text{c}}^{\max}\right)h}$ (S10)

The resistance change during unloading originates primarily from the contact resistance $R_{\text{c}}$ at the crack interface, governed by the disconnection–reconnection mechanism of the crack-face materials. ^[4]^ When the local crack width *w* is smaller than the height $h_{i}=k\varepsilon_{i}$ of a microscopic asperity, contact is established and current conducts across the crack. With the crack width during unloading scaling linearly with strain $w_{\text{c}}=k_{w}\cdot\varepsilon$, the normalized conductance at the crack is, in simplified form:

$S_{\text{c}}=\frac{\sum_{i} N_{i}\theta\left(\varepsilon_{i}-\varepsilon\right)}{\sum_{i} N_{i}}$ (S11)

where $\theta\left(\varepsilon_{i}-\varepsilon\right)$ is the Heaviside step function and Ni is the number of crack asperities with height $k\varepsilon_{i}$. Introducing the normalized probability distribution *p*(*ε*) of asperity sizes, Equation (S11) is rewritten as:

$S_{\text{c}}=\int_{\varepsilon}^{\varepsilon_{\max}} p(y)\text{d}y$ (S12)

According to the Greenwood–Williamson statistical contact-mechanics model, asperity heights follow an exponential distribution, causing the contact area to vary exponentially with separation distance; *p*(ε) is therefore taken as an exponential distribution function:

(S13)

where $k_{\text{s}}$ is a decay coefficient reflecting surface roughness; a larger $k_{\text{s}}$ indicates contact concentrated near *ε* = 0. From the normalization condition that *p*(*ε*) integrates to unity over (0, $\varepsilon_{\text{max}}$), one obtains $C=k_{\text{s}}/\left(1-e^{-k_{\text{s}}\cdot\varepsilon_{\max}}\right)$. Combining Equations (S12) and (S13) yields Equation (5) of the main text. Assuming the contact resistance at full crack closure is $R_{\text{c}}^{\left(0\right)}$, the crack resistance at strain *ε* is $R_{\text{c}}=R_{\text{c}}^{\left(0\right)}/S_{\text{c}}$.

During unloading, the number of formed cracks does not change; the total resistance is therefore:

$R=\left(n_{\max}+1\right)R_{\text{u}}^{(2)}$ (S14)

where $n_{\max}=N_{\text{sat}}[1-\exp(-k_{\text{n}}\cdot\varepsilon_{\max})]$ is the crack count at maximum strain. Arranging the unloading-phase relations and substituting into the definition of Δ*R*/*R*_0_ yields the unloading-phase governing equation, Equation (6) of the main text.

| $\left(\frac{\Delta R}{R_{0}}\right)_{\text{unload}}=\frac{lh}{\rho_{2}^{\left(0\right)}\text{L}}\left(n_{\max}\text{+1}\right)\frac{S_{\text{c}} R_{1}^{\left(2\right)}R_{2}^{\left(2\right)}\text{+}\left(R_{1}^{\left(2\right)}\text{+}R_{2}^{\left(2\right)}\right)R_{\text{c}}^{\left(0\right)}}{S_{\text{c}}\left(4R_{1}^{\left(2\right)}\text{+}R_{2}^{\left(2\right)}\right)\text{+}R_{\text{c}}^{\left(0\right)}}-1$ | Equation (6), main text |
| --- | --- |

**Note S4. Leave-One-Out Cross-Validation Comparison: Physics-Constrained Model vs. Radial-Basis-Function Response Surface**

To quantify the predictive contribution of the physics-based model structure developed in Section 3.4, we constructed an alternative response surface using radial basis function (RBF) interpolation — a purely data-driven approach that imposes no physical constraints on the functional form — and compared its leave-one-out cross-validation (LOOCV) accuracy against that of the physics-constrained model under identical conditions.

**S4.1 RBF Response-Surface Construction**

The RBF response surface was built from the same eight groups of steady-state electromechanical data (3–30 mm pre-stretch) used to calibrate the physics-constrained model. For each pre-stretch level, the mean steady-state response curve provides the mapping from working strain to normalized resistance change Δ*R/R*_0_. To handle the unequal strain ranges across pre-stretch levels (each group spans a different maximum strain dictated by its pre-strain amplitude), the data were assembled in the two-dimensional (pre-stretch, working strain) input space with Δ*R/R*_0_ as the scalar output. Boundary augmentation was applied identically to the physics-constrained model pipeline: the residual resistance was interpolated via PCHIP, and the peak resistance at each pre-stretch level was obtained by fitting the loading-phase electromechanical equation (Equation (3), main text) to the measured steady-state peaks.

**S4.2 Hyperparameter Optimization**

The RBF interpolant requires specification of a kernel function and associated hyperparameters. To ensure a fair comparison, we performed a systematic grid search over 19 candidate configurations spanning five kernel types (thin-plate spline, cubic, linear, multiquadric, and Gaussian) with varying smoothing parameters and shape factors. Each configuration was scored by its mean LOOCV root-mean-square error (RMSE) evaluated over the six interior groups (5, 10, 15, 20, 25, and 28 mm pre-stretch); the two boundary groups (3 and 30 mm) were excluded from the scoring to prevent data-sparse boundary behavior from biasing the hyperparameter selection toward boundary-specific tuning at the expense of interior fidelity. The optimal configuration — multiquadric kernel, smoothing parameter 1 × 10⁻⁵, shape factor ε = 1.0 (mean interior RMSE = 0.46) — was adopted for all subsequent evaluations.

**S4.3 LOOCV Evaluation**

With the optimized hyperparameters fixed, the full eight-group LOOCV was executed: in each of the eight rounds, one pre-stretch group was withheld entirely, the RBF response surface was reconstructed from the remaining seven groups (including re-interpolation of the residual resistance and re-fitting of the peak resistance), and a fully blind prediction was generated for the withheld group. The predicted response curve was then compared with the experimental data in physical space, and the coefficient of determination (*R*²) was computed. The identical procedure was applied to the physics-constrained model (Section 3.6, main text); Table S6 presents the side-by-side comparison.

**Table S6**. LOOCV *R*² comparison between the physics-constrained electromechanical model and the optimized RBF response surface. Bold entries highlight the higher R² for each withheld group.

| Withheld group (mm) | Physics-constrained model *R*² | RBF response surface *R*² |
| --- | --- | --- |
| 3 | 0.816 | -0.457 |
| 5 | 0.970 | 0.979 |
| 10 | 0.985 | 0.987 |
| 15 | 0.991 | 0.993 |
| 20 | 0.994 | 0.996 |
| 25 | 0.996 | 0.996 |
| 28 | 0.997 | 0.999 |
| 30 | 0.978 | 0.994 |
| Mean | 0.966 | 0.811 |

**S4.4 Discussion**

The comparison reveals a clear pattern. For interior design-space configurations (5–28 mm), where bilateral interpolation support is available, the optimized RBF surface achieves generalization accuracy comparable to — and in several cases marginally higher than — the physics-constrained model. This outcome is expected: in data-dense regions, a well-tuned interpolant can capture the smooth variation between calibration points without requiring physical constraints.

The decisive difference emerges at the design-space boundary. For the 3 mm group, the RBF prediction collapses to *R*² = −0.457 — a negative value indicating that the prediction is less accurate than a constant-mean baseline — whereas the physics-constrained model retains R² = 0.816, successfully tracking the experimental curve shape with only an offset in peak amplitude (Figure S8a, main text). This divergence arises because the RBF has no mechanism to constrain its output beyond the convex hull of the calibration data, whereas the physics-constrained model enforces asymptotic boundedness of the electromechanical response and a monotonic decay form for the crack-surface attenuation coefficient λ, both of which physically preclude the non-monotonic oscillations that afflict the unconstrained interpolant at the boundary.

The per-group RBF predictions are plotted in Figure S13, with the corresponding physics-constrained model predictions overlaid for visual comparison.

Figure S13. Per-group LOOCV predictions of the optimized RBF response surface

**Note S5. Uncertainty Mapping Procedure, Threshold Rationale, and Sensitivity Analysis**

**S5.1 From LOOCV Residuals to a Resolved Epistemic Error Metric**

The epistemic uncertainty quantifies the approximation error of the analytical model relative to the true physical process. At each of the eight calibration pre-stretch levels, this error is obtained from the LOOCV procedure of Section 3.6: the withheld specimen's blind-prediction curve $\hat{y}_{i}(\varepsilon)$ is compared point by point with the experimental data $y_{i}(\varepsilon)$, producing a spatially resolved error profile along the working-strain axis.

The raw point-wise absolute residual $r_{i}(\varepsilon_{j})=|\hat{y}_{i}(\varepsilon_{j})-y_{i}(\varepsilon_{j})|$ is noisy, so it is regularized by a centered sliding-window root-mean-square operator with an adaptive, odd window width $\tilde{w}_{i}=\max(3, \mathrm{round}(0.07 N_{i}))$, where *Nᵢ* is the number of experimental points of condition *i*. The local RMSE is then normalized by the peak experimental response $P_{i}=\max y_{i}(\varepsilon_{j})$ of that condition, yielding a dimensionless local NRMSE, denoted: $U_{\text{epi}}(\varepsilon_{j})=\frac{\text{RMSE}_{i}^{\text{loc}}(\varepsilon_{j})}{P_{i}}$.

This normalization is essential because the peak Δ*R*/*R*_0_ spans more than an order of magnitude across the eight pre-stretch levels (from 1.31 at 3 mm to 79.27 at 30 mm); without it, high-pre-stretch conditions would dominate the uncertainty field purely by virtue of their larger response amplitudes.

**S5.2 RBF Interpolation onto the Continuous Design Space**

The above procedure yields the local NRMSE along eight discrete pre-stretch lines — 4 080 scattered samples $\{(\varepsilon_{p,m}, \varepsilon_{m}), U_{\text{epi},m}\}$ in total — whereas the design space is continuous in the pre-stretch direction. To obtain a continuous field $U_{\text{epi}}(\varepsilon_{p}, \varepsilon)$, the scattered samples are interpolated with a thin-plate-spline radial basis function () together with a first-degree polynomial tail, under exact interpolation constraints (smoothing parameter = 0). The thin-plate spline minimizes integral bending energy among all interpolants, ensuring the smoothest surface consistent with the measured residuals and producing physically smooth transitions between adjacent pre-stretch lines.

The interpolant is evaluated on a boundary-conforming parametric grid: pre-stretch sampled at 0.5 mm displacement increments (55 levels), and for each pre-stretch the working strain sampled as $\varepsilon=u \varepsilon_{\max}(\varepsilon_{p})$ with $u\in[0, 1]$ (150 points per level). This parametric mesh keeps the grid aligned with the physically attainable envelope and avoids staircase artifacts at the domain boundary. Any small negative interpolation overshoot is clipped to zero.

The aleatoric uncertainty field $U_{\text{ale}}\left(\varepsilon_{p},\varepsilon\right)$ is constructed by the identical RBF procedure, with the experimentally measured cycle-to-cycle standard deviation of the steady-state response (error bars in Figure 3i of the main text) replacing the LOOCV-derived NRMSE as the input quantity.

**S5.3 Composite Uncertainty and Threshold**

The epistemic and aleatoric contributions are statistically independent and are combined by root-sum-of-squares composition into a total uncertainty $U_{\text{total}}=\sqrt{U_{\text{ale}}^{2}+U_{\text{epi}}^{2}}$. In the multi-objective optimization of Section 4.2, design configurations with $U_{\text{total}}$ > 0.05 are discarded as insufficiently reliable. This threshold corresponds to a maximum tolerable prediction error of 5% (NRMSE), a value consistent with typical instrumentation-grade accuracy requirements in strain-sensor calibration. The threshold is an engineering judgment rather than a theoretically derived quantity; its influence on the design outcome is therefore assessed by sensitivity analysis below.

**S5.4 Sensitivity of the Knee-Point Solution to the Uncertainty Threshold**

To evaluate whether the recommended pre-stretch configuration depends on the specific threshold value, the Pareto optimization of Section 4.2 was repeated with the uncertainty threshold varied from 0.03 to 0.10 while all other constraints (linearity *R*² ≥ 0.98, hysteresis *H* ≤ 2%) were held fixed. Table S7 summarizes the results.

**Table S7. Sensitivity of the knee-point solution to the composite uncertainty threshold.**

| Uncertainty threshold $U_{\text{total}}$ | Feasible Pareto solutions | Knee-point pre-stretch (mm) | Knee-point pre-strain (%) |
| --- | --- | --- | --- |
| 0.03 | 97 | 18 | 43.0 |
| 0.04 | 97 | 18 | 43.0 |
| 0.05 (baseline) | 97 | 18 | 43.0 |
| 0.06 | 97 | 18 | 43.0 |
| 0.08 | 97 | 18 | 43.0 |
| 0.10 | 97 | 18 | 43.0 |

Both the knee-point location and the number of feasible Pareto solutions are completely invariant across the entire tested range. This invariance arises because all 97 Pareto-optimal configurations that satisfy the linearity and hysteresis constraints already exhibit *U*_total_ well below the most restrictive threshold tested (0.03). In other words, the regions of the design space with favorable sensitivity–linearity–hysteresis trade-offs coincide spatially with the regions of highest model confidence and experimental reproducibility — a physically expected outcome, since moderate pre-strain levels that yield balanced performance also lie in the data-dense interior of the calibration range. The uncertainty threshold therefore functions as a safety net rather than an active filter in this problem: it would only become binding if the Pareto front extended into data-sparse boundary regions, which the linearity and hysteresis constraints have already excluded. The uncertainty metric exerts its design influence through its role as one of the four Pareto objectives — shaping the trade-off surface geometry and thereby the knee-point location — not through the threshold.

**Reference**

[1]	Jeon, H., Hong, S. K., Kim, M. S., Cho, S. J., Lim, G., "Omni-Purpose Stretchable Strain Sensor Based on a Highly Dense Nanocracking Structure for Whole-Body Motion Monitoring," *ACS Applied Materials & Interfaces* 9, no. 48 (2017): 41712,

https://doi.org/10.1021/acsami.7b14153.

[2]	Yamada, T., Hayamizu, Y., Yamamoto, Y., et al., "A stretchable carbon nanotube strain sensor for human-motion detection," *Nature Nanotechnology* 6, no. 5 (2011): 296, https://doi.org/10.1038/nnano.2011.36.

[3]	Shang, J.-C., Pan, W.-F., Zhao, W.-H., et al., "Optimizing patterned laser-induced graphene strain sensors via novel piezoresistive modeling and multi-objective analysis," *Chemical Engineering Journal* 519(2025): 165228, https://doi.org/10.1016/j.cej.2025.165228.

[4]	Kang, D., Pikhitsa, P. V., Choi, Y. W., et al., "Ultrasensitive mechanical crack-based sensor inspired by the spider sensory system," *Nature* 516, no. 7530 (2014): 222, https://doi.org/10.1038/nature14002.
